# A FRET-Based DNA Biosensor Tracks OmpR-Dependent Acidification of *Salmonella* during Macrophage Infection

**DOI:** 10.1371/journal.pbio.1002116

**Published:** 2015-04-14

**Authors:** Smarajit Chakraborty, Hideaki Mizusaki, Linda J. Kenney

**Affiliations:** 1 Mechanobiology Institute, National University of Singapore, Singapore; 2 Jesse Brown Veterans Affairs Medical Center, Chicago, Illinois, United States of America; 3 Department of Microbiology and Immunology, University of Illinois-Chicago, Chicago, Illinois, United States of America; HHMI, Massachusetts Institute of Technology, UNITED STATES

## Abstract

In bacteria, one paradigm for signal transduction is the two-component regulatory system, consisting of a sensor kinase (usually a membrane protein) and a response regulator (usually a DNA binding protein). The EnvZ/OmpR two-component system responds to osmotic stress and regulates expression of outer membrane proteins. In *Salmonella*, EnvZ/OmpR also controls expression of another two-component system SsrA/B, which is located on *Salmonella* Pathogenicity Island (SPI) 2. SPI-2 encodes a type III secretion system, which functions as a nanomachine to inject bacterial effector proteins into eukaryotic cells. During the intracellular phase of infection, *Salmonella* switches from assembling type III secretion system structural components to secreting effectors into the macrophage cytoplasm, enabling *Salmonella* to replicate in the phagocytic vacuole. Major questions remain regarding how bacteria survive the acidified vacuole and how acidification affects bacterial secretion. We previously reported that EnvZ sensed cytoplasmic signals rather than extracellular ones, as intracellular osmolytes altered the dynamics of a 17-amino-acid region flanking the phosphorylated histidine. We reasoned that the *Salmonella* cytoplasm might acidify in the macrophage vacuole to activate OmpR-dependent transcription of SPI-2 genes. To address these questions, we employed a DNA-based FRET biosensor (“I-switch”) to measure bacterial cytoplasmic pH and immunofluorescence to monitor effector secretion during infection. Surprisingly, we observed a rapid drop in bacterial **cytoplasmic** pH upon phagocytosis that was not predicted by current models. Cytoplasmic acidification was completely dependent on the OmpR response regulator, but did not require known OmpR-regulated genes such as *ompC*, *ompF*, or *ssaC* (SPI-2). Microarray analysis highlighted the *cadC/BA* operon, and additional experiments confirmed that it was repressed by OmpR. Acidification was blocked in the *ompR* null background in a Cad-dependent manner. Acid-dependent activation of OmpR stimulated type III secretion; blocking acidification resulted in a neutralized cytoplasm that was defective for SPI-2 secretion. Based upon these findings, we propose that *Salmonella* infection involves an acid-dependent secretion process in which the translocon SseB moves away from the bacterial cell surface as it associates with the vacuolar membrane, driving the secretion of SPI-2 effectors such as SseJ. New steps in the SPI-2 secretion process are proposed.

## Abstract 2

Rather than using the cadaverine operon to combat the acid conditions of the macrophage vacuole, Salmonella allows its cytoplasm to become acidified, which in turn drives secretion of effectors into the host cell.

## Author Summary

The human pathogenic bacteria *Salmonella* encounters extreme and diverse conditions during the course of host infection. Survival and adaptation inside the host requires highly regulated virulence factors. When *Salmonella* is engulfed by a macrophage, it forms a vacuole-type structure that is actively acidified by the macrophage in an attempt to kill or neutralize the bacteria. However, the acidic pH in this “*Salmonella*-containing vacuole,” or SCV, does not kill the bacteria and is instead a major inducing signal for the expression of a specific class of virulence proteins. These proteins, encoded in the *S* *almonella* Pathogenicity Island 2 (SPI-2), function as a nanomachine that mediates the delivery of *Salmonella* effector proteins that disrupt host immune defenses. In this paper, we investigate unanswered questions regarding the ability of Salmonella to survive the low pH and its consequences for bacterial growth in the SCV. Using a fluorescent biosensor, we monitored the intracellular pH of the *Salmonella* cytoplasm while it resides in the SCV during macrophage infection. Our results indicate that the bacteria cytoplasm acidifies in response to SVC acidity; this acidification requires the transcription factor OmpR, a known regulator of SPI-2. OmpR represses the *cadC/BA* operon, which is involved in the recovery from acid stress, thus enabling *Salmonella* to assume the acidic pH of the macrophage vacuole. Acidification is required for the secretion of virulence factors; blocking acidification resulted in a neutralized cytoplasm that was defective for SPI-2 secretion. Our work challenges existing views that bacteria regulate their pH to maintain neutrality, and provides a new model for *Salmonella* virulence factor secretion and infection.

## Introduction

Gram-negative pathogens use type III secretion systems (T3SS) to secrete effectors into the host, which promote virulence and alter host signaling functions. *Salmonella enterica* serovar Typhimurium encodes two T3SS on *Salmonella* pathogenicity islands 1 and 2 (SPI-1 and SPI-2). Their unique secreted effectors are primarily active during different phases of infection. SPI-1 effectors promote adherence and initial infection of the intestinal epithelium, while SPI-2 effectors are responsible for survival and replication in the macrophage vacuole [1–3] and bacterial spreading to distal organs [4]. The SPI-1 needle complex has been well characterized both functionally and structurally [5–7], but the SPI-2 needle complex is fragile and not very abundant and has not been well characterized. This raises questions about the conditions that induce SPI-2 needles during *Salmonella* infection and about how SPI-2 needles function. In the present work, we show that the *Salmonella* cytoplasm is acidified both in vitro and in vivo in response to acid stress. Furthermore, acidification is necessary for OmpR activation of SPI-2–dependent secretion, but not assembly. Thus, the macrophage vacuole provides signals that activate SPI-2 expression, assembly, and secretion in vivo, and these include acidification of the bacterial cytoplasm.

After entry into the macrophage, *Salmonella* resides in a modified intracellular compartment, the *Salmonella*-containing vacuole (SCV). Several studies indicate that the pH of this compartment is approximately 5 [8–10], and the SCV is comparable to a normal phagosome in terms of its biogenesis [11]. Once inside the vacuole, the EnvZ/OmpR two-component system is activated. In *Escherichia coli*, the EnvZ/OmpR system is a sensor of osmotic stress, regulating expression of outer membrane porins [12]. We recently discovered that the EnvZ histidine kinase responds to **cytoplasmic** osmotic stress rather than extracellular stress, and it is capable of sensing osmolality and activating its downstream target OmpR without being in the membrane [13]. Based on this result, we postulated that *Salmonella* might respond to the acidic pH of the macrophage vacuole by acidifying its cytoplasm, providing the protons that drive formation of the activated conformation of EnvZ, which promotes phosphorylation and phosphotransfer to OmpR [13]. Thus, we set out to measure the cytoplasmic pH of *Salmonella* while it resides in the SCV. For these experiments, we employed a novel DNA biosensor (the “I-switch”) that undergoes non-Watson-Crick base pairing in the presence of excess protons, resulting in fluorescence resonance energy transfer (FRET) [14,15]. We tested the I-switch in *Salmonella* in vitro and then used I-switch–containing bacteria to infect RAW264.7 macrophages. This study represents the first application of the I-switch in which it has been used to measure the unknown pH of an intracellular compartment. Our results indicate that in vitro, *Salmonella* acidifies its cytoplasmic compartment in response to extracellular acid stress, and during infection, the *Salmonella* cytoplasm rapidly acidifies in response to the low pH of the vacuole in which it resides. Thus, the bacterial cytoplasm responds positively to extracellular (vacuolar) pH changes. Furthermore, EnvZ and OmpR are completely required for acidification, establishing OmpR as a regulator of genes that enable *Salmonella* to survive the acid stress of the SCV. The known, well-characterized targets of OmpR regulation, including OmpC, OmpF, and SPI-2 [16–18] were not required for acidification, indicating that new, unidentified OmpR targets were involved. Microarray analysis in the absence and presence of *ompR* at pH 5.6 indicated that approximately 390 genes were up-regulated. Of interest was the cadaverine operon *cadC/BA*, which is involved in recovery from acid stress. In the macrophage vacuole, these genes were repressed by OmpR and the *Salmonella* cytoplasm remained acidified. In the absence of *ompR*, *Salmonella* regulated its intracellular pH and returned to near neutral in a CadC/BA-dependent manner. This activation of EnvZ/OmpR by acid stress stimulates production of the SsrA/B two-component system (located on SPI-2), and ultimately, expression of SPI-2-secreted effectors [16–18].

During *Salmonella* infection, bacteria switch from assembling structural components of the T3SS (represented herein by the translocon SseB) to secreting effectors (SseJ in this study) into the host cytosol to modify host functions. We used the I-switch to test an existing model that proposed a neutralization step was required for the switch from translocon secretion to effector secretion. Our results indicate that when the vacuole is not acidified, effector secretion does not occur, in agreement with some previous results [19] but differing from others [20]. Interestingly, we found that the onset of SseJ effector secretion correlated with an outward movement of the SseB translocon away from the bacterial cell surface. This outward movement coincided with an association of SseB with the vacuolar membrane. This step required vacuolar acidification, since the presence of the V-ATPase inhibitor Bafilomycin prevented vacuolar acidification and SseB remained on the *Salmonella* cell surface. As a result, effector secretion into the host cytosol was prevented. Our results suggest a new model where acidification of the bacterial cytoplasm drives the secretion and release of translocon protein(s) from the bacterial cell surface, which in turn facilitates effector secretion.

## Results

### The I-switch Tracks Intracellular pH in *Salmonella*

**Fig 1:**
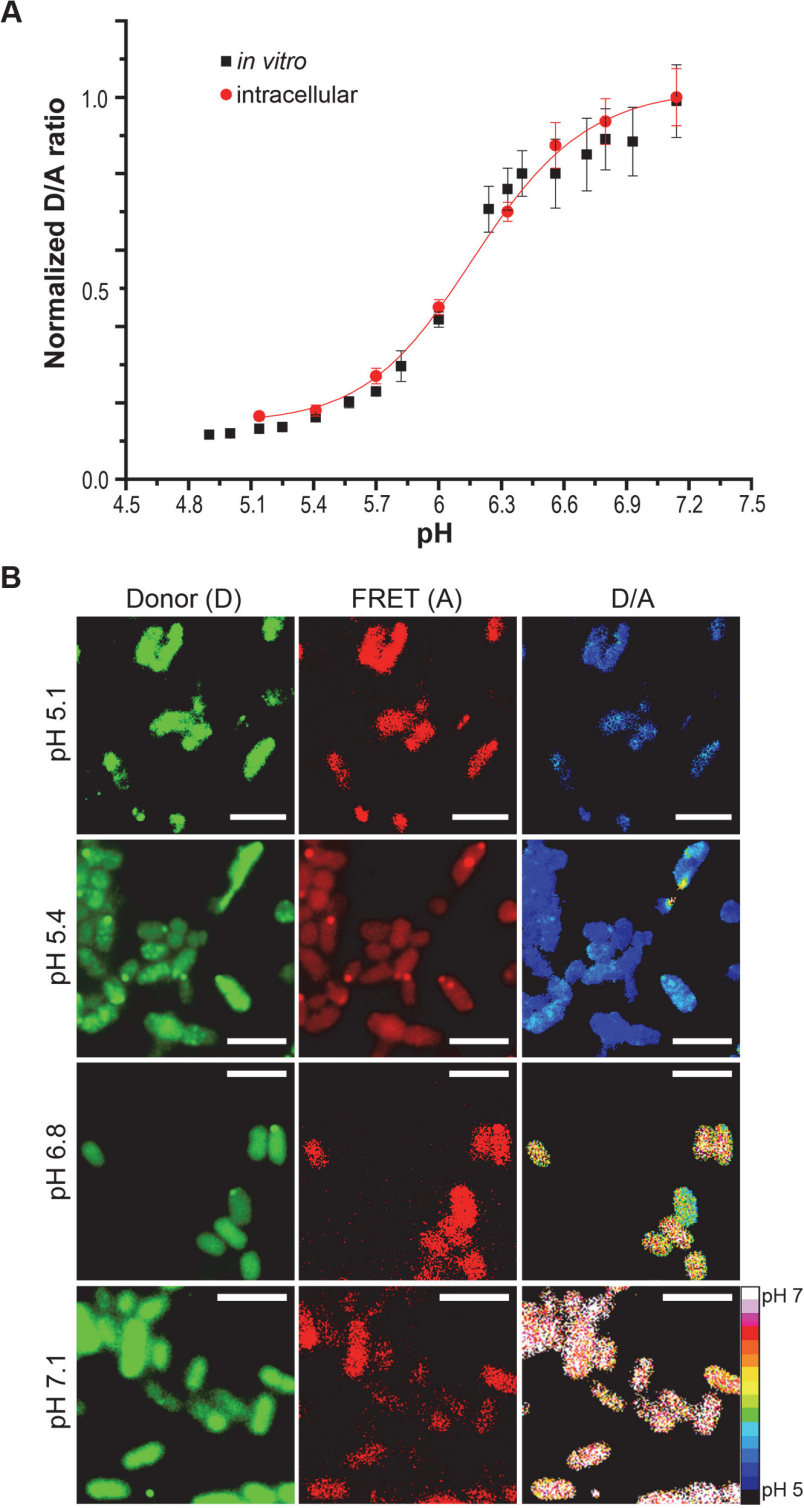
Functional integrity of the I-switch in *Salmonella*. Wild-type (WT) *Salmonella* was electroporated with 6 μM I-switch and recovered in SOC for 1 h at 37°C, followed by three washes with PBS. Bacteria were then clamped at various pH values in K^+^-rich buffer containing 40 μM nigericin for 1 h at room temperature (RT) and imaged on an Applied Precision Delta Vision inverted wide-field fluorescence microscope. The fluorescence intensity of both donor and acceptor channels of 50 cells were measured for every pH value indicated in the figure. The ratio of D/A intensities were obtained and the normalized D/A ratios were plotted as a function of pH, which yielded a sigmoidal intracellular calibration curve (**A**). The intracellular curve is overlaid on the in vitro calibration curve. Error bars represent mean ± standard deviation (SD). **(B)** Representative Donor (D), FRET (A) and D/A ratio images of WT *Salmonella* clamped at various pH values. Scale bar, 3 μm, color scale, bottom, right. Pseudocolor images were generated by calculating the D/A ratio per pixel. Using ImageJ software, the pixels were then color-coded using 16 colors with blue (D/A = 0.1) to red (D/A = 1) to indicate the transition from acidic to neutral pH.

To determine the impact of acid stress on *Salmonella* intracellular pH (pH_i_), we used the I-switch (I_A488/A647_) labeled at its 5′ and 3′ termini with Alexa-488 and Alexa-647, respectively, as a cytoplasmic pH sensor. The I–switch consists of cytosine-rich unpaired regions that form an anti-tetraplex CH^+^.C by alternate Watson and Crick base pairing in the presence of protons. This leads to a “closed” conformation of DNA, enabling FRET to occur between the fluorophore pairs. This process is reversible, and at neutral pH, the I-switch dissociates into an open, extended conformation because of electrostatic repulsion between the duplex arms [14,15]. We first compared the fluorescence intensity of the donor alone (520 nm, "D") and the acceptor alone (666 nm, "A") with the fluorescence of the I-switch containing both donor and acceptor. This provided a determination of the cross-talk between the fluorophores, which was negligible (S1A Fig). I-switch labeled with Alexa 488 and Alexa 647 was suspended in buffers ranging from pH 5.0 to 7.2. The samples were excited at 480 nm and spectra were recorded in a Tecan spectrophotometer (GENios) from 510 to 700 nm. A D/A curve as a function of pH was plotted from the ratio of donor (520 nm) to acceptor (666 nm) intensities to generate the in vitro standard curve (Fig 1A). The I-switch exhibited a sigmoidal increase over this range due to the formation of the I-motif in the closed state.

We next investigated the ability of the I-switch to function inside bacterial cells by incorporating I_A488/A647_ into *Salmonella* by electroporation and verifying its incorporation by fluorescence microscopy (Fig 1B). We confirmed its intracellular location by preparing spheroplasts, which eliminated the outer membrane but still retained the I-switch in the cytoplasm (S1B Fig). We used 6 μM I_A488/A647_ for electroporation, since it showed optimum fluorescence at both donor and acceptor channels inside bacterial cells; this concentration was used in all subsequent experiments. The internal pH of *Salmonella* was clamped to the external pH of K^+^-containing media using 40 μM nigericin [21–23]. An intracellular D/A standard curve (Fig 1A) showed perfect correlation with the in vitro curve, indicating the functionality and integrity of the I-switch in *Salmonella*. The D/A profiles of cells clamped at acidic pH 5.1 were visibly distinct and showed a 5-fold increase from those clamped at neutral pH 7.1. This study is the first application of the I-switch in bacteria.

### Extracellular Acid Stress Acidifies the *Salmonella* Cytoplasm and Requires EnvZ/OmpR Activation

**Fig 2:**
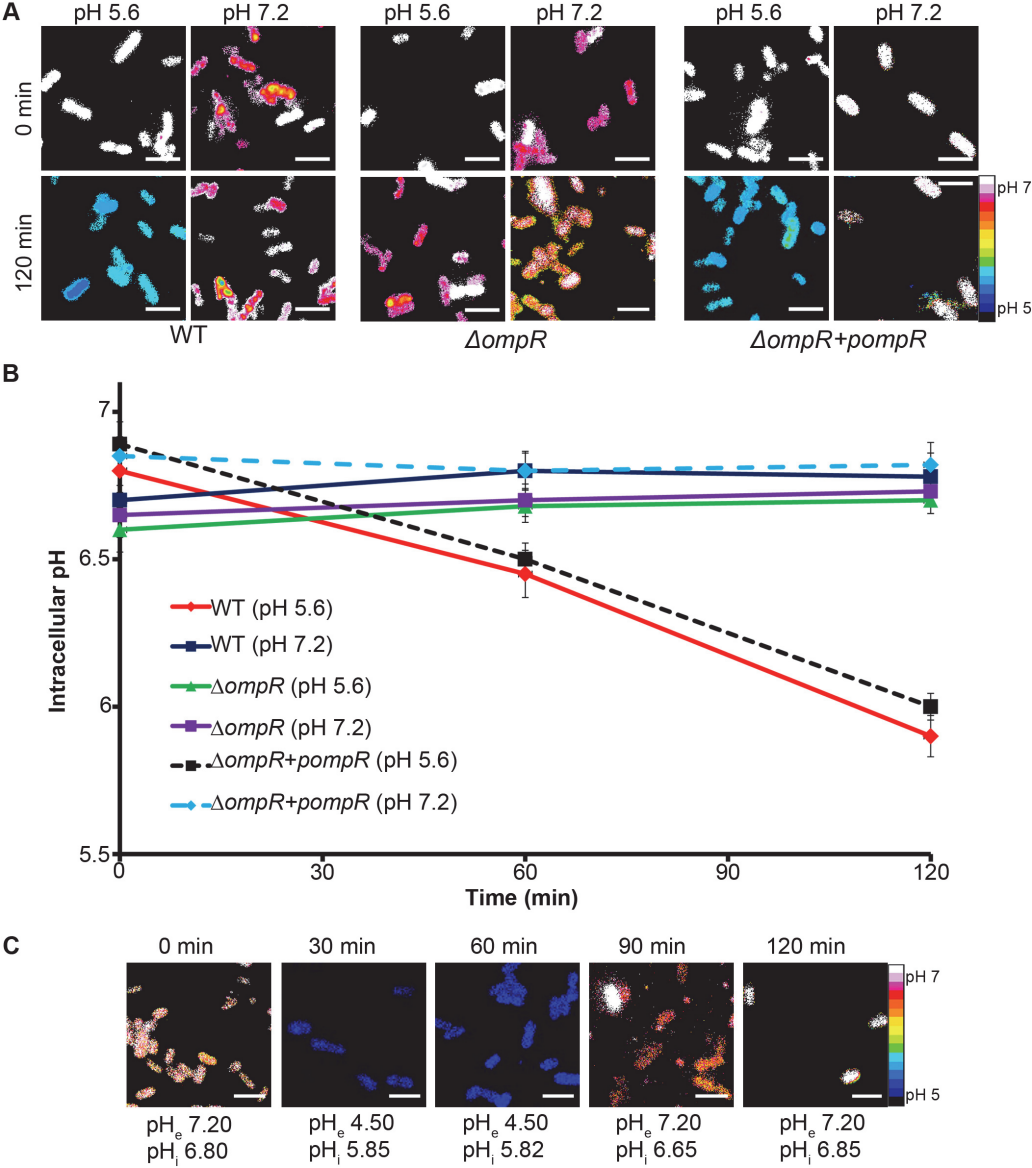
The *Salmonella* cytoplasm is acidified upon acid stress and requires OmpR. **(A)** *Salmonella* cultures of I_A488_/I_A647-_incorporated WT, *ompR* null mutant, and *ompR* null complemented with pWSK29-*ompR* (encoding *ompR*) were incubated at either acidic pH_e_ (5.6) or neutral pH_e_ (7.2) at indicated time points. Representative epifluorescence of the D/A ratio images are shown for WT *Salmonella*, the *ΔompR* mutant, and the *ΔompR* mutant with *ompR* provided in *trans*. Scale bar, 3 μm. **(B)** A plot of the intracellular pH of *Salmonella* WT at pH_e_ 5.6 and pH_e_ 7.2, an *ompR* null mutant at pH_e_ 5.6 and pH_e_ 7.2, and the *ompR* null mutant complemented with *ompR* supplied in *trans* at pH_e_ 5.6 and pH_e_ 7.2 over time. The D/A ratios of 50 cells were analyzed at each time point and the pH values were determined from the intracellular standard curve. Error bars represent mean ± standard error of the mean (SEM) (*n* = 3). (**C**) To demonstrate the reversibility of the I-switch and mimic vacuolar pH conditions in vitro, WT *Salmonella* cultures were incubated at pH_e_ (4.5) for 1 h, and samples were collected at 30 and 60 min for imaging. The cultures were then washed and incubated for an additional 1 h at pH_e_ 7.2. The D/A ratios of 50 cells were analyzed at the indicated time points. Representative epifluorescence of the D/A ratio images are shown. Scale bar, 3 μm. The average pH_i_ from 50 cells is listed under the image.

We next measured the change in cytoplasmic pH (pH_i_) in response to varying the pH of the extracellular media (pH_e_). *Salmonella* containing the I-switch were subjected to acid stress and the fluorescence was measured in SPI-2–inducing (pH_e_ 5.6) and non-inducing (pH_e_ 7.2) conditions over time (Fig 2A). The pH_i_ of *Salmonella* decreased from 6.85 to 6.1 upon incubation at pH_e_ 5.6, but remained fairly constant when incubated at pH_e_ 7.2 (Fig 2B), indicating that the *Salmonella* cytoplasm acidifies in response to extracellular acidic pH. Addition of extracellular media at even lower pH_e_, e.g., 4.5, reduced the intracellular pH further, to pH 5.8 (Fig 2C). Acidification was readily reversible upon addition of extracellular media at alkaline pH (Fig 2C), which restored the near neutrality of the cytoplasm. This result further demonstrates the functionality of the I-switch; i.e., it is not degraded, because it can reversibly switch from an open state (low FRET at neutral pH) to a closed state (high FRET at acidic pH) in response to changes in pH_e_.

**Fig 3:**
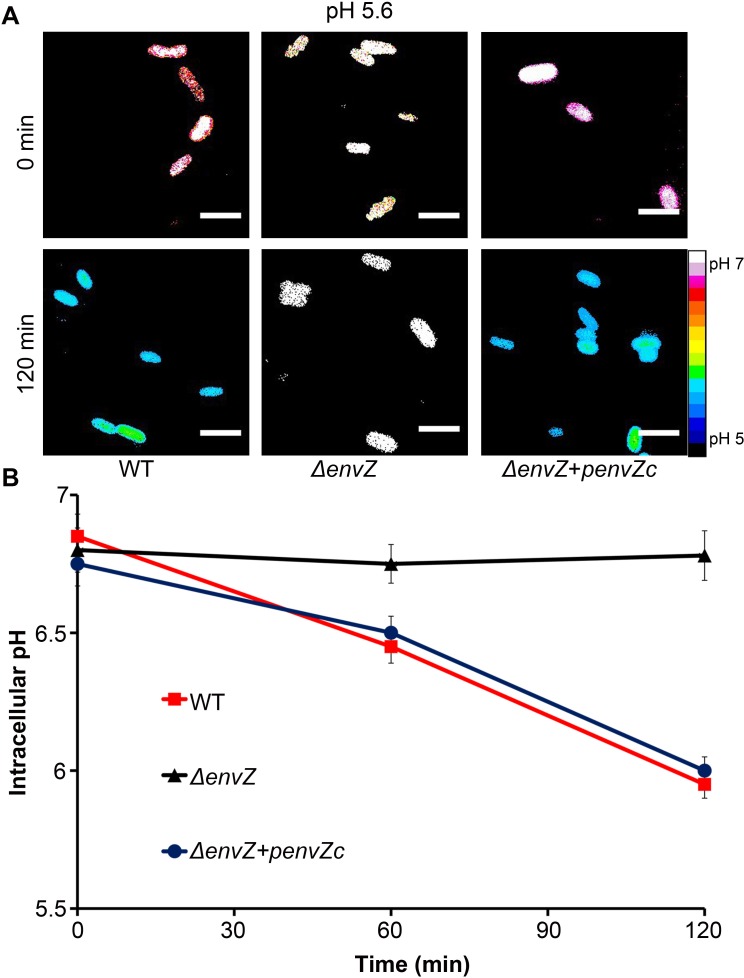
The cytoplasmic domain of EnvZ is required for intracellular acidification upon acid stress. **(A)** *Salmonella* cultures of I_A488_/I_A647-_incorporated WT, *envZ* null mutant, and *envZ* null complemented with pMPM-*envZc* (encoding cytoplasmic domain of *envZ*) were incubated at acidic pH_e_ (5.6) at indicated time points. Representative epifluorescence of the D/A ratio images are shown. Scale bar, 3 μm. **(B)** A plot of the intracellular pH at pH_e_ 5.6 of *Salmonella* WT, an *envZ* null mutant and the *envZ* null mutant complemented with *envZc* supplied in *trans* over time. The D/A ratios of 50 cells were analyzed at each time point and the pH values were determined from the intracellular standard curve. Symbols represent the mean ± SEM (*n* = 3).

Because OmpR was implicated in the Acid Tolerance Response in *Salmonella* ([24]; see also S2 Fig), we measured the response of an *ompR* null strain of *Salmonella* under similar acid stress conditions. In the *ompR* null strain, the intracellular pH did not decrease in response to extracellular pH stress (Fig 2A and 2B). OmpR-dependent intracellular acidification was completely restored upon supplying *ompR* in *trans* on a plasmid (Fig 2A and 2B). This result provides the first evidence for a role of OmpR as a regulator of acid stress in *Salmonella* by regulating genes that alter intracellular pH. In addition, the *envZ* null strain did not respond to acid stress, but acidification was restored upon supplying the cytoplasmic domain of *envZ* (*envZc*) in *trans* (Fig 3A and 3B). The requirement of the cytoplasmic domain of the EnvZ kinase only (EnvZc) further indicates that the I-switch is in the cytoplasm and the cytoplasmic pH becomes acidic under these conditions. Acidification was not dependent on growth phase, but was similar at both exponential and stationary phase (S3 Fig).

### 2',7'-Bis-(2-Carboxyethyl)-5-(and-6)-Carboxyfluorescein, Acetoxymethyl Ester (BCECF-AM) Reports Similar Acidification As the I-switch in Response to Extracellular Acid Stress

To further ensure that the I-switch was a high-fidelity reporter of intracellular pH, we compared it to the fluorescent probe BCECF-AM, which has been widely used to measure intracellular pH in mammalian cells [25,26], plant cells [27], yeast [28,29], and bacteria [23,30,31]. It allows ratiometric detection (S4A and S4B Fig) and is permeable to bacterial membranes. Once it gains access to the intracellular compartment, it is cleaved by intracellular esterases to create the active fluorescent form. Although the range of BCECF-AM (pH 6–7) is more limited than the I-switch (pH 5.1–7.1), a similar acidification of the *Salmonella* cytoplasm was observed in response to extracellular acid stress (S4C Fig), corroborating the use of the I-switch in reporting the pH of the *Salmonella* cytoplasm. Our observation that BCECF (which is intracellular) and the I-switch reported similar intracellular pH values corroborates the intracellular location of the I-switch, and the I-switch was also clearly visible in spheroplasts (S1B Fig).

### Cytoplasmic Acidification Occurs Immediately After Entry into the Macrophage SCV

**Fig 4:**
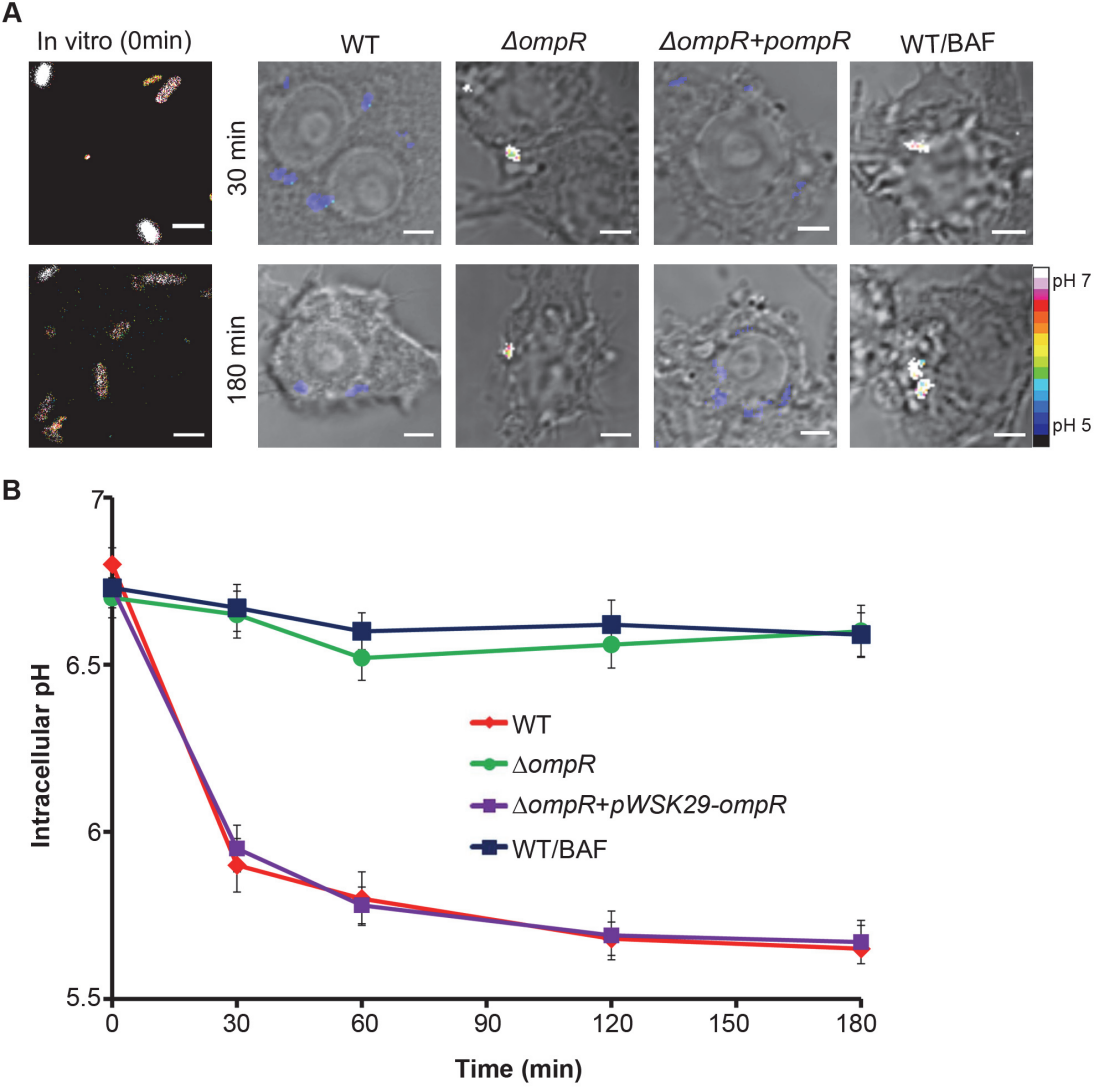
The *Salmonella* cytoplasm is rapidly acidified upon macrophage infection and requires OmpR. RAW264.7 macrophages infected with WT *Salmonella* and the *ΔompR* null mutant containing I_A488_/I_A647_ were imaged over time on a Nikon A1R confocal microscope. The D/A ratio in the *ΔompR* strain showed minimal FRET, indicating *ompR* was required for cytoplasmic acidification. RAW264.7 macrophages were pre-treated with 25 nM of the V-ATPase inhibitor bafilomycin A1 (BAF) for 30 min prior to infection, and macrophages were infected with I_A488_/I_A647-_electroporated *Salmonella* in the presence/absence of BAF for 3 h and imaged as before. BAF neutralized the SCV and prevented *Salmonella* acidification. **(A)** Representative images of D/A ratios are shown at 30 and 180 min post-infection and compared to WT *Salmonella* prior to infection. Infection was carried out as described in Materials and Methods. Scale bar, 3 μm. **(B)** Prior to infection, the D/A ratios of *Salmonella* were determined and the intracellular pH was approximately 6.8. The intracellular pH of *Salmonella* remained fairly constant in macrophages upon BAF treatment (approximately 6.8) or in the *ΔompR* null strain in macrophages, unlike untreated WT *Salmonella*. It showed a remarkable intracellular pH drop to 5.65, similar to the *ompR* null mutant complemented with *ompR* supplied in *trans*. Dataset is represented as the mean ± SEM (*n* = 3).

Because *Salmonella* acidified its cytoplasm in response to external acid stress (Figs 2 and 3), we reasoned that uptake of *Salmonella* into the acidified SCV should also lead to a decrease in intracellular pH. This internal acidification would in turn provide a signal to EnvZ/OmpR to induce the SPI-2 type III secretion system by activating SsrA/B [16,18], which is critical for the survival of *Salmonella* within the phagocyte. To measure the intracellular pH of *Salmonella* during infection in RAW264.7 macrophages, we used confocal microscopy to directly visualize I-switch containing *Salmonella* inside macrophages. At each time point, the D/A ratio of individual cells was determined as a measure of intracellular pH (Fig 4). *Salmonella* exhibited a remarkable, rapid intracellular pH drop (6.85 to 5.75) immediately post-infection and reached a plateau value of 5.65 after 3 h. Interestingly, the *Salmonella ompR* null mutant did not experience a decrease in intracellular pH upon entry into the SCV (Fig 4). Again, the dependence of *ompR* on cytoplasmic acidification could be fully complemented by supplying *ompR* in *trans* (Fig 4). This result identifies OmpR as a key regulator of *Salmonella* intracellular pH, not only for in vitro acid stress but also during macrophage infection. The I-switch containing bacteria in the vacuole were viable because they were capable of SPI-2 secretion (see below).

An alternative method employed by many *Salmonella* researchers for examining intracellular bacteria involves Triton solubilization at various time points post-infection to recover bacteria from macrophages. Although this method is less rigorous than direct visualization by confocal microscopy, the results obtained using this approach were in agreement with the microscopy (S5 Fig). The pH*i* of cells recovered after solubilization was determined from the reference intracellular standard curve. The pH*i* of *Salmonella* rapidly dropped by nearly 1 unit (from 6.81 to 5.87) immediately within 30 min post-infection and reached a final value of 5.6 at 3 h post-infection. Bacteria recovered from the vacuole were capable of growth on agar plates, further indicating that they were still viable. Taken together, both approaches firmly establish that uptake of *Salmonella* into the SCV leads to an OmpR-driven immediate drop in intracellular pH. This result has significant ramifications in interpreting the pH control of the gating of the SPI-2 type III secretion apparatus [20] (see Discussion).

### Inhibition of Vacuolar Acidification by Bafilomycin Prevents *Salmonella* Acidification

Phagosomal pH is maintained between 4 to 5 by the vacuolar H^+^-ATPase (V-ATPase) and remains acidified for at least 5–6 h post-infection [9,32]. Treatment with the specific inhibitor Bafilomycin A1 (BAF) prevents SCV acidification [1]. To examine the contribution of vacuolar SCV acidification to the low intracellular pH of *Salmonella* during macrophage infection, we used BAF, which is known to raise the pH of endosomes to neutral in both HeLa [33] and RAW264.7 cells [19]. In the presence of BAF, the *Salmonella* cytoplasm remained approximately pH 6.7 throughout the infection and failed to acidify (Fig 4). The pH_i_ in the presence of BAF was similar to the *ompR* null mutant. Our results with BAF clearly indicate that the acidic pH of the SCV leads to a decrease in intracellular pH of *Salmonella* in macrophages (Fig 4). Pre-treatment with and maintenance of 25 nM BAF throughout infection completely abrogated the intracellular pH decrease in *Salmonella*-infected macrophages. This effect was rapidly reversible [34], as removal of BAF after 1 h restored the ability of *Salmonella* to reduce intracellular pH, suggesting a complete correlation between vacuolar acidification and an intracellular pH drop (S6 Fig). Together, these data provide strong evidence that the cytoplasm of *Salmonella* rapidly follows the pH of the macrophage vacuole and requires OmpR (Fig 4).

### Well-Characterized OmpR-Regulated Genes Were Not Responsible for Intracellular Acidification

Because OmpR regulates the SsrA/B two-component system that, in turn, activates the type III secretion system encoded by SPI-2 [16–18,35,36], we wanted to determine whether or not SPI-2 was the target that was essential for cytoplasmic acidification. We performed a similar analysis on a mutant *Salmonella* strain that does not make a type III secretory apparatus. An *ssaC* null strain lacks the gene encoding the outer membrane ring protein SsaC of the SPI-2 type III secretion system, and thus is not capable of type III secretion. The *ssaC* null strain was fully capable of cytoplasmic acidification, as shown in (S7A and S7B Fig). This was not surprising, because we observed a similar OmpR-dependent response to external acid in *E*. *coli*, which lacks SPI-2 (unpublished results). OmpR is best characterized for its regulation of the outer membrane proteins OmpF and OmpC [12]. An *ompC* mutant and the double *ompC*/*ompF* mutant lacking the genes encoding the outer membrane proteins OmpC and OmpF were similarly capable of acidification (S7A and S7B Fig). A previous study identified MgtC as an inhibitor of the FoF1 ATPase, leading to acidification [37], but an *mgtC* null strain was as acidified as the WT (S7A and 7B Fig). Thus, OmpR regulates an unidentified target(s) that is responsible for cytoplasmic acidification.

### OmpR Directly Represses *cadC/BA* to Acidify the *Salmonella* Cytoplasm

**Fig 5:**
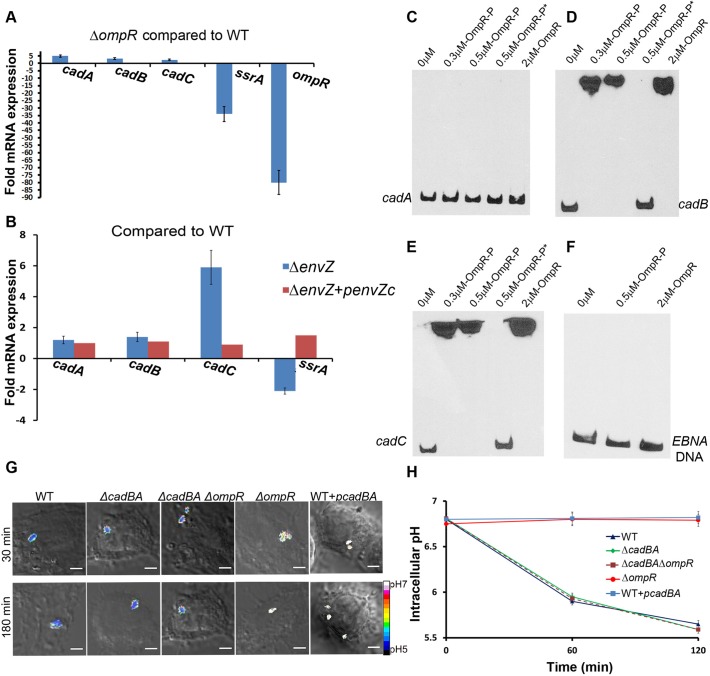
OmpR directly represses *cadC/BA* to prevent recovery from acidification in the macrophage vacuole. **(A)** mRNA levels of *cadA*, *cadB*, *cadC*, *ssrA*, and *ompR* genes were determined by quantitative real-time polymerase chain reaction (qRT-PCR) from WT and *ompR* null strains as described in Materials and Methods. The error bars represent the mean ± SD (*n* = 6). The *ompR* null strain showed increased expression of *cadC/BA* genes, indicating OmpR repression. **(B)** mRNA levels of *cadA*, *cadB*, *cadC*, and *ssrA* genes were determined by qRT-PCR from WT, *envZ* null mutant and the *envZ* null mutant complemented with *envZc* as described in Materials and Methods. The error bars represent the mean ± SD (*n* = 6). The *envZ* null strain showed increased expression of *cadC/BA* genes, indicating a role for EnvZ in OmpR-mediated repression. The cytoplasmic domain of *envZ* alone could restore *ssrA* expression levels when provided in *trans*, indicating that it was responding to cytoplasmic signals. **(C–F)** Electrophoretic mobility shift assays (EMSA) experiments were conducted to examine the interaction between OmpR and *cadC/BA*. OmpR was incubated with 10 fmol of biotin end-labeled *cadA* **(C)**, *cadB* **(D)**, *cadC* **(E)**, along with 60 bp biotin end-labeled EBNA DNA **(F)** as a negative control. Addition of 50-fold excess unlabeled DNA is indicated by an asterisk. **(G)** Representative images of D/A ratios are shown at 30 and 180 min post-infection of WT, the *cadBA* double null mutant, an *ompR/cadBA* triple null strain and a *cadBA* over-expressed strain of *Salmonella*. Infection is described in Materials and Methods. Scale bar, 3 μm. **(H)** Plot of the D/A ratios of various mutants of *Salmonella*. The D/A ratio of approximately 30 cells were calculated at the indicated time points post-infection. Symbols represent the mean ± SEM (*n* = 3).

In order to identify genes that were regulated by OmpR, we performed a microarray analysis and compared the transcriptome of the *ompR* null and the WT strain under both acid and osmotic stress. We identified genes belonging to the *cadC/BA* operon to be highly up-regulated in the *ompR* mutant. We validated the microarray results by investigating the expression of the *cadC/BA* operon under acid stress by quantitative real-time PCR. Transcripts for genes *cadC* (2.3-fold), *cadB* (3.2-fold) and *cadA* (4.9-fold) were all up-regulated in the *ompR* mutant, suggesting that OmpR functions as a repressor at this locus (Fig 5A). Known targets of OmpR activation, including *ssrA* and *ompR* (*ompB*), were highly down-regulated in the absence of *ompR* (Fig 5A). The *ssrA* transcript was down-regulated in the *envZ* null strain; it was restored to the WT levels upon supplying *envZc* in *trans*. This result further demonstrates that OmpR activation is via EnvZc (Fig 5B). The *envZ* null strain showed higher expression of *cadA* (1.2-fold), cad*B* (1.4-fold), and *cadC* (5.9-fold) and failed to acidify its cytoplasm upon acid stress (Fig 3).

To examine whether OmpR repression was a result of a direct interaction, we performed electrophoretic mobility shift assays (Fig 5C–5F). DNA sequences upstream of *cadC* (-354 to -3), upstream of *cadB* (-362 to -1) and 264-bp upstream to 34-bp downstream of *cadA* relative to the ATG in each case were amplified using primers that were biotinylated on the 5′-ends. The resulting biotinylated DNA fragment was used in the assay with purified OmpR or OmpR~P prepared by phosphorylation from acetyl phosphate (Fig 5C) [38]. The formation of an OmpR~P-DNA complex is clearly visible in the presence of 0.3 μM OmpR~P at both *cadB* and *cadC* promoters (Fig 5D and 5E). The addition of 50-fold excess of unlabeled *cadB/C* DNA resulted in the release of the labeled probes (Fig 5D and 5E, OmpR~P*), confirming that the complexes formed were due to a specific interaction of OmpR~P with DNA. Neither OmpR nor OmpR~P bound to a 60 bp biotin end-labeled Epstein-Barr Nuclear Antigen **(**EBNA) DNA, a control for non-specific binding (Fig 5F). In the absence of phosphorylation, OmpR bound to DNA (Fig 5D and 5E), but with considerably lower affinity [38]. Interestingly, no binding was evident at *cadA* (Fig 5C), suggesting *cadB* and *cadA* may be co-transcribed. Thus, OmpR represses *cadC/BA* by directly binding to upstream DNA at *cadC* and *cadB*.

We next measured the intracellular pH of the *cadBA* null strain in the macrophage vacuole and compared it to the WT and *ompR* null strains (Fig 5G and 5H). If OmpR functions as a repressor of *cadC/BA* (Fig 5A), we would expect the *cadBA* null strain to be similar to the WT strain in terms of cytoplasmic acidification, i.e., knocking out *cadBA* would be the same as OmpR repressing *cadBA*. Indeed, the pH_i_ of the *cadBA* null strain was similar to WT *Salmonella* and unlike the bafilomycin-treated strain or the *ompR* null strain (see Figs 4, 5G and 5H). This result identifies the *cadC/BA* operon as the major system that eliminates protons upon acid stress and maintains pH homeostasis when *Salmonella* is in the macrophage vacuole. OmpR represses the *cadC/BA* system to prevent recovery from acidification. When the *cadBA* genes are not present, the presence of OmpR is not required for acidification (Fig 5G and 5H), indicating a key role for OmpR in repressing the *cad* system in the vacuole. This acidification is required for activation of SPI-2 effector expression and secretion (see below).

Over-expression of *cadBA* was able to completely over-ride the OmpR-dependent acidification, and the *Salmonella* cytoplasm remained neutral both during macrophage infection and in response to in vitro acid stress (Fig 5G and 5H; S8 Fig). The pH was similar to an *ompR* null strain (Fig 2B) or to BAF-treated cells (Fig 4), clearly confirming that *cadBA* enables the *ompR* mutant to restore pH homeostasis in the macrophage vacuole. The *cadBA* null strain acidified more rapidly than the WT strain, presumably because it takes some time for OmpR to repress the *cad* system (S8 Fig). However, the intracellular pH was nearly identical to the WT strain by 120 min.

### OmpR Up-Regulates atpB, Increasing Intracellular ATP Levels

The F_1_F_o_ ATP synthase employs the proton gradient to synthesize ATP [39]. Because the *Salmonella* cytoplasm was acidified when grown at pH_e_ 5.6, we anticipated cytosolic ATP levels would be higher than at neutral pH. Indeed, switching *Salmonella* from pH 7.2 to pH 5.6 increased intracellular ATP levels 1.8-fold compared to the *ompR* null strain (S9A Fig). ATP was restored to WT levels when the *ompR* null strain was complemented with *ompR* supplied in *trans*. Control experiments with an *atpB* null strain or in the presence of the uncoupler carbonyl cyanide m-chlorophenyl hydrazone (CCCP) drastically reduced intracellular ATP. In our microarray, ATP synthase genes were down-regulated in the absence of *ompR* by 2- to 3-fold. We then compared the mRNA levels of *atpB*, a gene encoding the *a* F_o_ subunit, by qRT-PCR in the WT and *ompR* null strains. The transcript for *atpB* was up-regulated (2.1-fold) in the WT strain, suggesting a role for OmpR in activation of *atpB* (S9B Fig). Thus, OmpR appears to play a dual role in acid stress, by activating proton translocation via increased F_1_F_o_ ATP synthase coupled with elimination of proton consumption via repression of the *cad* system. Taken together, these combined effects enable WT *Salmonella* to reduce intracellular pH upon acid stress.

### Acidification Leads to SseB Translocon Movement Away from the Salmonella Cell Surface

**Fig 6:**
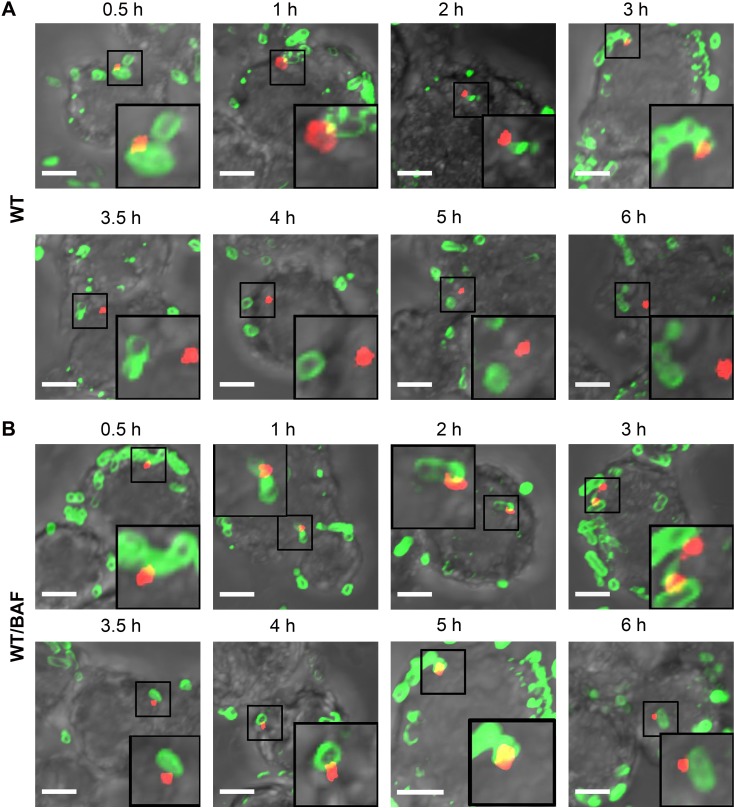
The translocon protein SseB is released from the *Salmonella* surface over time, and acidification is required. **(A)** WT *Salmonella* were electroporated with unlabeled I-switch, recovered, and used for infection in RAW264.7 macrophages for various indicated times. 25 nM BAF **(B)**, or an equal volume of DMSO, was used to pre-treat the macrophages for 30 min prior to infection and was maintained throughout the infection time. The cells were fixed, permealized, and stained for *Salmonella* LPS (green) and translocon protein SseB (red). Scale bar, 3 μm.

**Fig 7:**
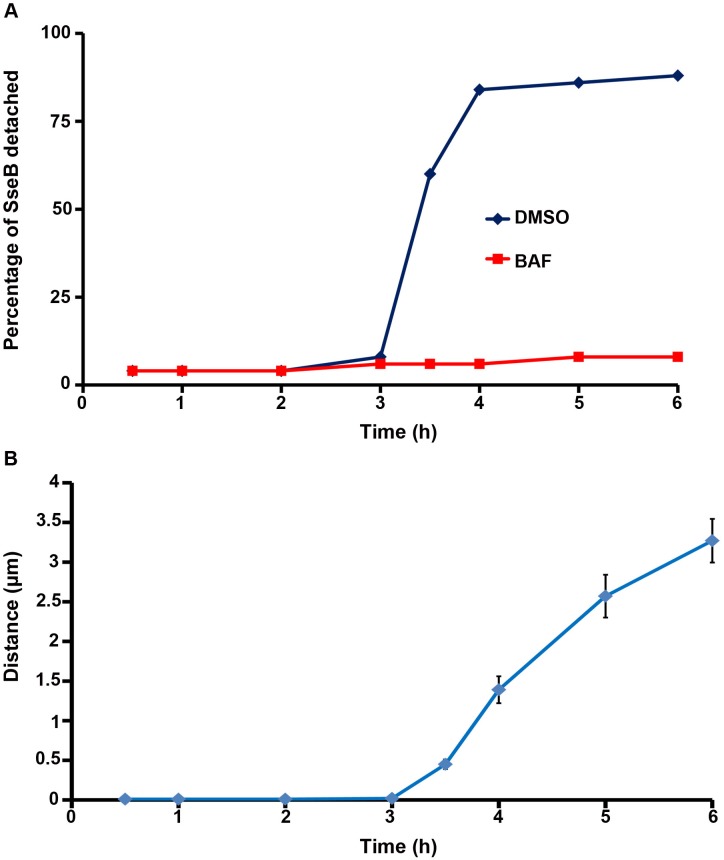
The percentage of detached SseB increased over time upon acidification. **(A)** The percentage of detached SseB was plotted over time post-infection as described in the text. **(B)** The distance (in μm) of 50 SseB protein spots from the nearest bacterial cell surface was plotted as a function of time post-infection. Symbols are represented as the mean ± SEM.

We next investigated the kinetics of translocon secretion by immuno-labeling SseB in macrophages at various times post-infection. *Salmonella* were visualized using anti-LPS antibody up to 6 h. SseB was detectible immediately 30 min post-infection at one locus of the cell, and the number of SseB-labeled cells increased over time. After 3.5 h, SseB was no longer associated with a majority of *Salmonella* cells, but was removed from the cell surface (Figs 6A and 7). SseB was also detected in *Salmonella-*infected macrophages treated with BAF, but SseB remained on the bacterial cell surface (Figs 6B and 7A).

### Measurement of SseB from the *Salmonella* Cell Surface

To measure the extent to which SseB protein was separated from the bacterial cell surface, the Distance Transformation function of Imaris Software (version 7.7.1) was employed. The distance of the red SseB translocon protein to the nearest green *Salmonella* cell was measured by applying the distance transformation on the green channel surface to generate a new channel whose intensity indicated the shortest distance to the surface object border. Hence, for each red SseB protein spot, the minimum distance to its nearest green cell was obtained. For each time point post-infection, the distance of 50 SseB protein spots from the nearest cells were measured. A distance value of 0 indicated that SseB was attached to the cell surface, whereas a distance value larger than 0 indicated that SseB was separated from the surface. The percentage of distant SseB was plotted over time post-infection (Fig 7A). Using this approach, it was evident that the vast majority of SseB (>60%) were no longer surface-associated by 3.5 h, and by 6 h post-infection this value increased to 88% (Fig 7A). When acidification was prevented by treatment with BAF, the number of cells in which SseB was detached was only 5%–10%. Measurements of the distance (in μm) of SseB from the bacterial cell surface as a function of time post-infection are shown in (Fig 7B). At 6 h post-infection, the average distance from the *Salmonella* cell surface was approximately 3 μm, indicating that SseB was no longer cell-surface associated.

### SseB Co-localizes with the SCV Membrane after it Separates from the Bacterial Surface

**Fig 8:**
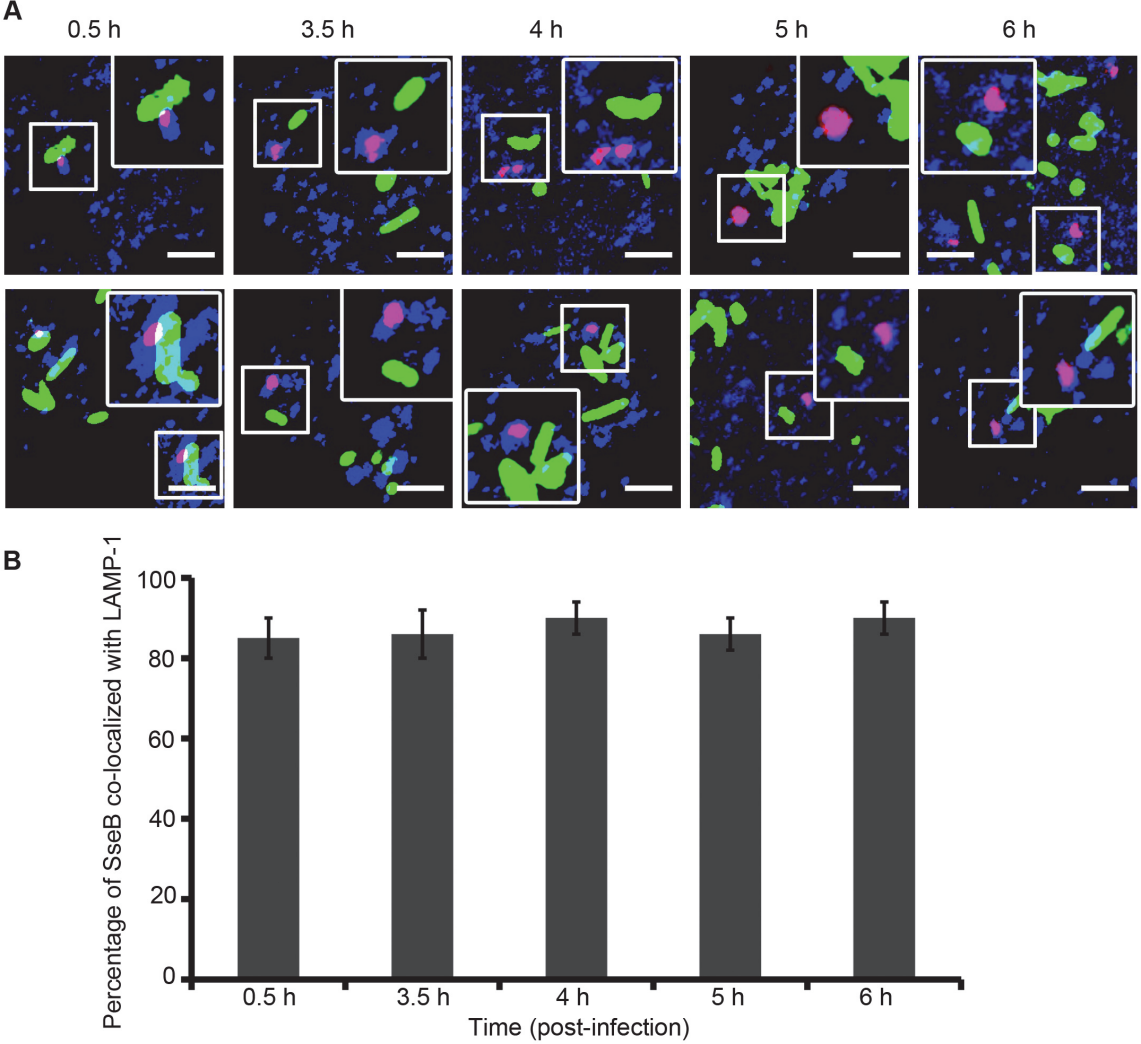
As SseB moves away from the *Salmonella* surface, it is associated with the vacuolar membrane. **(A)** RAW264.7 macrophages were infected with WT *Salmonella* harboring pFPV25.1 for the constitutive expression of GFP (green). Cells were fixed, permeabilized, and immunostained for mouse monoclonal anti-LAMP-1 (blue) and rabbit polyclonal anti-SseB (red) followed by Alexa568- or Alexa647-labeled secondary antibodies, respectively, for various times as indicated. The merged image indicates that once SseB was detached from the bacterial cell surface, it was co-localized with the host endosomal membrane (i.e., LAMP-1–positive membranes). Images were obtained using the Nikon A1R confocal microscope. Localization resulted in magenta staining of *Salmonella* SseB as analyzed by Image J software. Scale bar = 3μm. **(B)** Results of three independent experiments in which macrophages were infected with WT GFP-expressing *Salmonella* and immunostained with LAMP-1 and SseB at 3.5 h, 4 h, 5 h, and 6 h post-infection. The values represent the mean ± SEM.

*Salmonella* translocated effectors (SifA, SseF, SseG, PipB, and SpiC) localize to the SCV and to its tubular membranous structures termed *Salmonella*-induced filaments (Sifs) [40–42]. The Sifs extend from and connect SCVs during *Salmonella* replication inside host cells [43]. Therefore it was possible that once SseB was separated from the cell surface, it might be in association with endosomal tubules of vacuolar membrane origin. To address this question, we used immunofluorescence to compare the localization of SseB with the endosomal membrane marker, LAMP-1. RAW macrophages were infected with *Salmonella* WT harboring pFPV25.1 for constitutive expression of GFP and immunolabeled for LAMP-1 (blue) and SseB (red). Confocal microscopic images indicated that when SseB was separated from the *Salmonella* surface, it was co-localized with LAMP-1 positive membranes, resulting in magenta staining of SseB (Fig 8A). Nearly 90% of the translocon protein SseB was co-localized with LAMP-1 by 3.5 h and this level did not change up to 6 h post-infection (Fig 8B).

In the macrophage vacuole, a single focus of SseB was evident on the bacterial surface (Fig 6). This was similar to the localization of SseB that we observed in vitro (S10A Fig, bottom panel). Other components of the T3SS exhibited similar patterns of localization, including the outer membrane ring protein SsaC (top panel) and the inner membrane ring protein SsaJ (middle panel). Thus, the SPI-2 needle complex is localized primarily near to one end of the cell pole, unlike SPI-1 needles, which are distributed all over the bacterial surface [6,44]. An *ssaC* null strain was deficient for secretion and SseB was retained in the cytoplasm, substantiating that the focal assembly of the SPI-2 needle complex was competent for SPI-2 effector secretion (S10B Fig). Our observation that only approximately 13% of the population expressed SPI-2 needles on their surface in vitro highlights that, in contrast to SPI-1, SPI-2 is not abundant.

### Acidification Leads to SseJ Effector Secretion After SseB Translocon Surface Release

**Fig 9:**
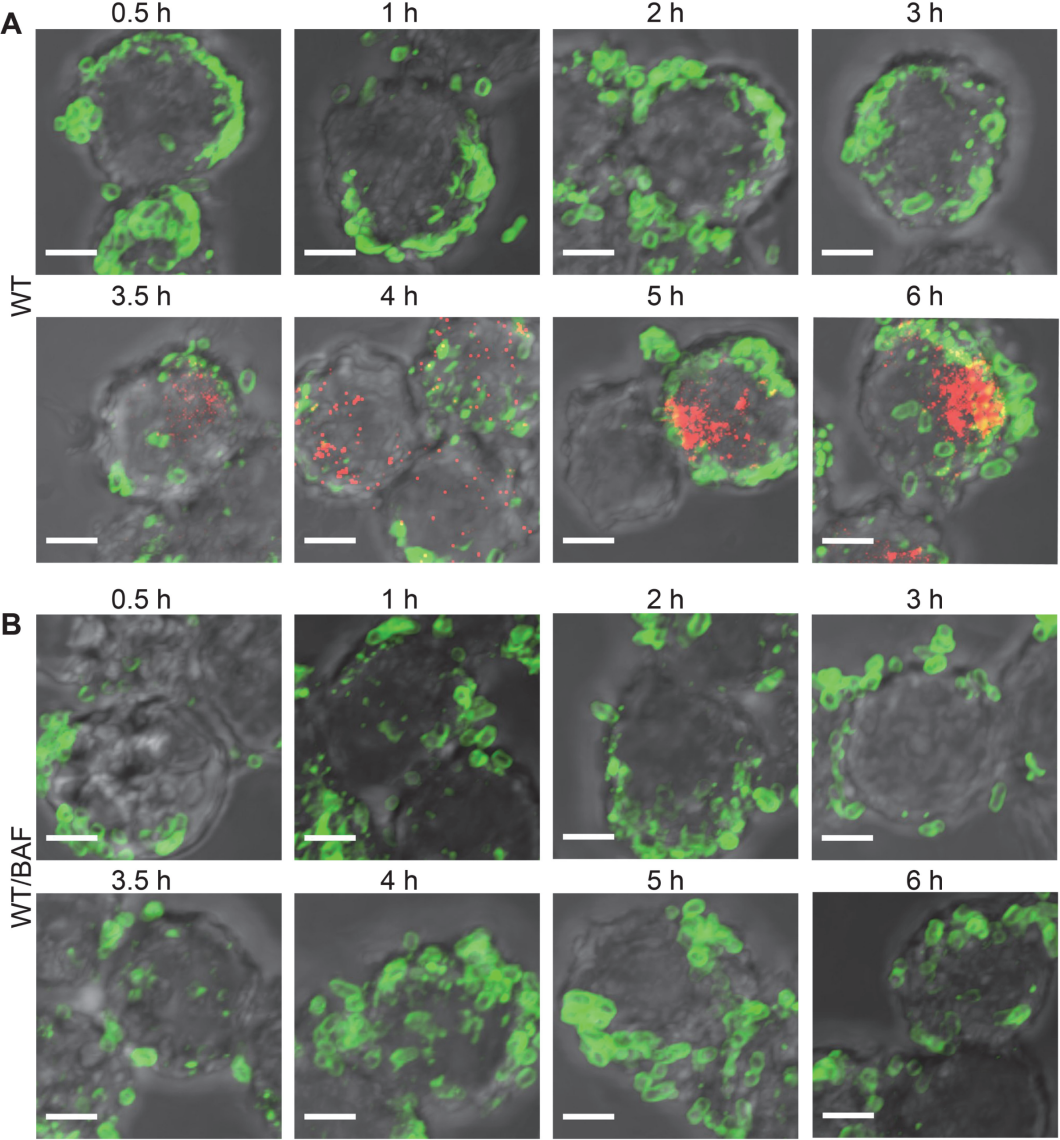
Acidification is required for secretion of effector SseJ. **(A)** RAW264.7 macrophages were infected with I-switch-electroporated *Salmonella* WT harboring p*sseJ-HA* for expression of SseJ-HA. Cells were immunostained for *Salmonella* LPS (green) and HA epitope (red), followed by Alexa488- or cy5-labeled secondary antibodies, respectively, for various time points as indicated. **(B)** 25 nM BAF or an equal volume of DMSO **(A)** was used as a control. Samples were imaged by confocal microscopy and analyzed using ImageJ software. Scale bar, 3 μm. SseJ secretion was observed (red puncta) after SseB release from the surface after 3.5 h.

To gain insight into the fate of effector secretion, we also compared the kinetics of SseJ secretion in *Salmonella*-infected macrophages by immunofluorescence microscopy. A strain expressing SseJ-HA was completely comparable to WT in terms of its intracellular pH response and replication in macrophages. SseJ-HA secretion was first detected 3.5 h post-infection in macrophages, similar to a time course of SseF-HA expression in HeLa cells reported previously [20]. The percentage of cells secreting SseJ increased between 3.5 and 6 h post-infection (Fig 9, visible as red puncta in the macrophage cytoplasm). In contrast to SseB secretion (Fig 6A), SseJ was not translocated in the presence of BAF, indicating that cytoplasmic acidification was essential for effector secretion (Fig 9B, lower panel; i.e., no red puncta are visible in the BAF-treated macrophages). SseJ secretion was subsequent to SseB release from the bacterial surface (Fig 6A and 6B). These results are in agreement with previous results [19] but significantly differ from other studies that reported that a neutralization step from the host was required for SPI-2 effector secretion [20]. Our results indicate that formation of the translocon pore is essential and precedes effector secretion. To verify that the translocon complex was required for effector secretion, we monitored the kinetics of SseJ secretion in an *sseB* null strain expressing SseJ-HA. No translocation of SseJ was evident, even after 6 h post-infection in the macrophages in the absence of SseB (S11 Fig).

## Discussion

Nucleic acids have been employed as remarkable tools to create synthetic nanomachines like DNA tweezers, walkers, and DNA-hybridization motors [45,46]. We describe herein a successful application of an artificially designed nanomachine in *Salmonella* to determine pH values under relevant physiological conditions that were previously unknown. The I-switch was effectively taken up by *Salmonella* after electroporation with ≥80% transformation efficiency (S12 Fig). However, at later time points, the limitation of the I-switch was apparent upon bacterial cell division, which resulted in dilution of I-switch positive cells (S12 Fig). In the macrophage vacuole, bacterial cell division is slower and the I-switch is effective for longer periods. Nevertheless, in the present study, using the I-switch, we measured a rapid acidification of the bacterial cytoplasm within 30 min upon entry into the SCV, followed by a further decrease to pH_i_ 5.65 over 3 h of infection.

Our results present evidence of **cytoplasmic** acidification of *Salmonella* inside the SCV during macrophage infection. This pH drop is a result of SCV acidification upon *Salmonella* infection [9,11,47]. Since the pH of the SCV remained below 5 during 6 h post-infection [9], and in the present study we show that the *Salmonella* cytoplasm mirrors vacuolar pH, we argue that intracellular acidification persists during the phase of macrophage infection when the translocon and effectors are being secreted. Both enumerated bacteria from macrophages and intracellular bacteria within the SCV reported similar intracellular pH values (Fig 4; S5 Fig). Our results go beyond measuring the pH of the SCV and link vacuolar pH to acidification of the *Salmonella* **cytoplasm**.

Our results differ substantially from studies in *E*. *coli* in suspension that reported an initial drop in intracellular pH after extracellular acid stress, followed by a rapid recovery within minutes [48]. More recent experiments in single cells on poly (L-lysine)-coated coverslips reported that 2%–23% of individual cells failed to recover from pH_i_ 5.5 [49], indicating significant stochastic variation. Current experiments underway in our laboratory indicate fundamental differences between *Salmonella* and *E*. *coli* (Chakraborty and Kenney, manuscript in preparation). Previous reports have shown that bacterial recovery times varied depending on the composition of the media and required potassium, presumably to stimulate K^+^-dependent proton efflux [48,50]. This rapid response was shown to require the *cadBA* operon [51], which would lead to a rapid neutralization. However, we found no evidence of K^+^-dependent intracellular neutralization in *Salmonella* (Chakraborty and Kenney, manuscript in preparation). Our experiments emphasize the role of EnvZ/OmpR in controlling intracellular *Salmonella* pH inside the SCV during macrophage infection. The intracellular pH of both *envZ* and *ompR* null mutants were not responsive to in vitro acid stress (Figs 2 and 3). In *Escherichia coli*, the OmpR/EnvZ two-component regulatory system plays a pivotal role in the modulation of gene expression in response to changes in extracellular pH [52,53]. The OmpR/EnvZ system is essential for *Salmonella* replication and survival within macrophages ([18]; see also S13 Fig) and to render full virulence in mice [54]. The *ompR* mutant strain showed a replication defect in macrophages, similar to that of SPI-2 deficient *ssaC* null strain (S13 Fig). To verify that intracellular acidification was essential for replication and survival within macrophages, we infected RAW264.7 macrophages with the *cadBA* over-expressed strain of *Salmonella* and monitored its ability to survive and replicate. Indeed, the *cadBA* over-expressed strain showed a similar replication defect as the SPI-2–deficient *ssaC* null strain. This result suggests that intracellular acidification increases the replication and survival fitness of *Salmonella* during macrophage infection.

Recently, it was shown that the inner membrane protein MgtC binds to AtpB, the *a* subunit of the F_1_F_o_ ATP synthase, inhibiting proton translocation and ATP synthesis [37]. In our experiments, the *mgtC* null strain was completely comparable to the WT in terms of its intracellular pH response (S7 Fig). The *mgtC* null strain exhibited an increase in intracellular ATP levels (S9A Fig), in agreement with previous reports [37]. Under our experimental conditions, *mgtC* transcripts were identical in both the WT and *ompR* null strains (S9B Fig), eliminating a role for OmpR in regulating MgtC [55]. These results suggest that different signaling pathways (e.g., EnvZ/OmpR versus PhoQ/PhoP) may play significant roles under different, unique activating conditions.

Our previous structural analysis of OmpR determined the physical basis for the ability of OmpR to function as a global regulator [56]. OmpR binds to AT-rich DNA, making phosphate backbone contacts, but very few base contacts [56]. Furthermore, OmpR contacts can vary at different promoters, i.e., DNA contacts are different at the porin genes compared to the SPI-2 *ssrA* gene [56]. This property of OmpR enables it to become a regulator of horizontally acquired genes during the course of evolution and to play a significant role in the response to acid stress. A previous study in *E*. *coli* reported that switching from aerobic to anaerobic respiration was important for surviving acid stress and also demonstrated a key role for OmpR in this switch, although no direct targets were identified [53]. The identified genes lacked complete OmpR binding sites, although OmpR binding sites are notoriously degenerate and difficult to define [38,56]. This lack of specificity makes OmpR a good global regulator, because it does not require specific amino acid/base contacts.

The SPI-2 T3SS is essential for survival of *Salmonella* within phagocytes [1,3], by avoiding the terminal stages of the degradative pathway [57]. Acidic pH and other environmental factors play an important role in the induction of SPI-2 genes [18,35,47,58,59]. Co-localization of *Salmonella* with the lysosome-associated membrane protein 1 (LAMP-1), clearly indicated that I-switch-incorporated *Salmonella* were in the SCV within 30 min after infection in macrophages and remained so during 6 h of macrophage infection (S14 Fig). Thus, the acidified cytoplasm of *Salmonella* is a result of residing in the acidified SCV and is one trigger of the onset of virulence gene expression. This view is substantiated by our results with BAF (Fig 4; S6 Fig), which demonstrated that blocking SCV acidification inhibited SPI-2 secretion.

**Fig 10:**
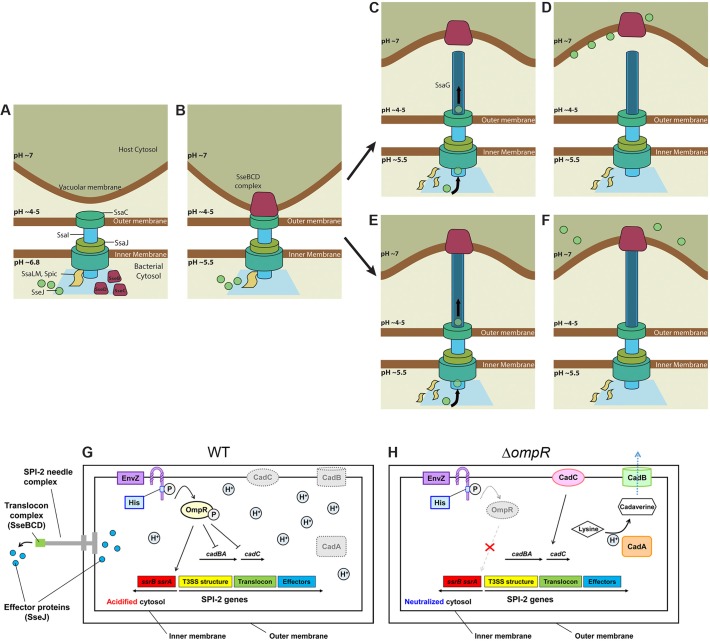
Model for SPI-2 secretion in macrophages. **(A)** Immediately post-infection, the bacterial cytosol is approximately pH 6.8 and the SPI-2 secretion apparatus is beginning to be assembled at the cell pole. **(B)** Within 30 min inside the SCV, the bacterial cytoplasm becomes acidic and the translocon complex SseBCD begins to assemble on the bacterial surface. The “**plug” model (C)** After 3.5 h, SseB is detached from the *Salmonella* surface into the macrophage cytoplasm, triggering the onset of SseJ effector secretion. **(D)** The bacterial cytoplasm remains acidified during the effector secretion process. **(E) The “SsaG needle elongation” model**: steps 1 and 2 are the same as in **(A, B)**. The SseBCD translocon complex begins to associate with the vacuolar membrane and “pulls” the SsaG needle, elongating the needle and driving the translocon away from the bacterial surface. **(F)** The pore opens and SseJ and other effectors are secreted. **(G)** At low external pH levels in the macrophage vacuole, protons (H^+^) cross the *Salmonella* outer and inner membrane and lower the pH of the cytoplasm. In WT *Salmonella*, EnvZ is activated by excess protons in the cytoplasm, activating OmpR. OmpR~P represses *cadC/BA* in the macrophage vacuole and the cytoplasm remains acidified. OmpR~P goes on to activate expression of SsrA/B, and SsrB~P activates SPI-2 transcription. **(H)** In the *ompR* null strain, CadC/BA is expressed and is responsible for amino acid decarboxylation. This process consumes intracellular protons, restoring cytoplasmic pH and maintaining intracellular pH homeostasis. Image credit: Chun Xi Wong.

SseB, SseC, and SseD form the translocon complex; SseC and SseD are membrane proteins, while SseB is soluble. Initially, SseB is on the bacterial cell surface inside the vacuole. Over time (approximately 3.5 h in our experiments), SseB moves further away from the *Salmonella* surface and becomes associated with LAMP-1 positive host membranes (Figs 6–9). This event coincides with pore-opening for effector secretion and might be dependent on vacuolar membrane contact. It is logical to assume that this separation occurs as the SsaG needle elongates, emerging from the SsaC ring in the outer membrane (Fig 10A, 10B, 10E and 10F). In the absence of acidification, SseB was not released from the bacterial surface and effector secretion did not occur. Neither SseC nor SseD were detected in RAW264.7 cells infected with an *sseB* null mutant *Salmonella* [19], confirming an important role of SseB in forming the translocon complex. Furthermore, secretion of SseJ was not observed in RAW264.7 cells infected with an *sseB* null *Salmonella* strain, indicating that translocon pore formation is also essential for effector secretion. The observation that effector secretion commences approximately 3.5 h post-infection suggests that this is the time required for formation of the translocon pore spanning the vacuolar membrane. Our attempts to tag the needle filament protein SsaG have thus far been unsuccessful but are a focus of current research efforts. Alternately, the translocon complex may detach from the SPI-2 needle, remaining in the vacuolar membrane, and providing a pore for SPI-2 effectors (Fig 10A–10D). Acidification leads to OmpR-P repression of the *cadC/BA* operon, resulting in cytoplasmic acidification of WT *Salmonella* (Fig 10G). In the *ompR* null strain, up-regulated CadC/BA consumes intracellular protons maintaining intracellular pH homeostasis (Fig 10H).

Acidification was absolutely required for the secretion of effector SseJ in macrophages (Fig 9). Our results are in agreement with some previous studies [19], but differ significantly from others [20], in that we never observe a neutralization step in the *Salmonella* cytoplasm prior to effector secretion. In summary, we used a novel biosensor and determined that the *Salmonella* cytoplasm acidifies immediately upon entry into the macrophage vacuole. This acidification was dependent on OmpR repression of the *cadC/BA* operon, and drives translocon release and subsequent effector secretion.

## Materials and Methods

### Cell Culture, Bacterial Strains, and Antibodies

RAW 264.7 cells were obtained from the American Type Culture Collection (ATCC, Manassas, VA, United States) and grown in a humidified 37°C, 5% CO_2_ tissue culture incubator. Cell culture reagents were from Life Technologies (Carlsbad, CA, US) unless otherwise stated. RAW 264.7 were maintained in Dulbecco’s Minimal Essential Media (DMEM) containing 2 mM L-glutamine, 1 mM sodium pyruvate and 17.5 mM D-glucose with 10% heat-inactivated fetal bovine serum (FBS). RAW 264.7 cells were always cultured in antibiotic-free media. Low passage number cells (<15 after receipt from ATCC) were seeded in 24-well dishes (Nunclon) and grown O/N before infection. The cells were washed gently with PBS and removed from the flask by scraping. The cells were suspended at a density of 1 × 10^5^ cells per well in 24-well plates. WT *S*. enterica serovar Typhimurium 14028s and the *ompR* null derivative (*ΔompR*) were used for all infection experiments unless otherwise indicated. DMSO was obtained from Sigma-Aldrich (St Louis, MO). To determine the acid stress response, bacterial strains were grown as in [60] in a modified N-minimal medium (MgM) buffered with 20 mM Tris (pH 7.2) or 20 mM MES (pH 5.6) containing 7.5 mM (NH_4_)_2_SO_4_, 5 mM KCl, 0.5 mM K_2_SO_4_, 1 mM KH_2_PO_4_, 10 mM MgCl_2_, 2 mM glucose, and 0.1% Casamino acids. The following antibodies were used for Immuno-labeling experiments: rabbit polyclonal anti-SseB, rabbit monoclonal anti-HA (Sigma), mouse monoclonal anti-*Salmonella* LPS (Abcam), rabbit anti-LAMP-1 (Abcam), mouse Alexa-488 IgG (Invitrogen), rabbit Alexa-488 IgG (Invitrogen), and rabbit cy5 IgG (Invitrogen).

### Transformation of the I-switch in *Salmonella*

I-switch sample preparation was performed as previously described [14] with the following modifications: 50 μM O1, O2, and O3 were mixed in equimolar ratios in 20 mM potassium phosphate buffer at the desired pH containing 100 mM KCl. Primers were annealed using touch-down PCR with a decrease in temperature of 1°C per 3 min from 95°C to 25°C and equilibrated overnight at 4°C before use. A concentration of 6 μM of I-switch DNA was used to transform electro-competent *Salmonella* cells. After electroporation, cells recovered for 1 h at 37°C in Super Optimal Broth (SOB) containing 20 mM glucose and 10 mM MgCl_2_ (SOC) unless otherwise stated. Cells were washed three times with PBS (Life technologies) and used for analysis.

### pH Clamping of *Salmonella*

pH clamping protocols were modified based on [14]. Double-labelled I-switch (I_A488/A647_) was diluted to 60 nM in clamping buffers (120 mM KCl, 5 mM NaCl, 1 mM MgCl, 1 mM CaCl, 20 mM HEPES buffered at various pH). The samples were excited at 488 nm and emission collected between 500–750 nm in a Tecan Spectrophotometer (GENios). The fluorescence intensity at 520 nm (D) was divided by the fluorescence intensity at 666 nm (A) and the D/A ratio was plotted over the pH range. For in vivo pH clamping experiments, bacterial cells electroporated with the I-switch were incubated in a K^+^-rich clamping buffer at various pH at RT for 1 h in the presence of 40 μM nigericin. Cells were placed on microscope slides (Marienfeld, Germany) coated with 1% agarose and then imaged by wide field fluorescence microscopy. Three independent measurements were recorded for each pH value and the D/A ratios were plotted as the mean ± SEM.

### *Salmonella* Infection in RAW264.7 Macrophages

*Salmonella* were first electroporated with the I-switch as described. Cells were seeded onto overnight-grown monolayers of RAW264.7 cells at a multiplicity of infection of 100:1 in 24-well plates. The plates were centrifuged at 500 rpm for 10 min to synchronize the infection. The infection was conducted for 30 min at 37°C in 5% CO_2_. Cells were washed three times with PBS and incubated with DMEM containing 100 μg/ml of gentamicin for 1 h at 37°C. Cells were again washed three times with PBS and incubated in DMEM containing 10 μg/ml gentamicin for the remainder of the experiment. For BAF-treated cultures, RAW264.7 cells were pre-treated with 25 nM BAF for 30 min prior to infection and 25 nM BAF was maintained in every step of the infection. At the stated time points, the cells were fixed using 3% PFA for 15 min at RT. The coverslips were mounted on a drop of gold anti-fade reagent (Invitrogen) and sealed with entellan (Merck). Samples were imaged using confocal microscopy (Nikon A1R) and analyzed by Image J imaging software. To inhibit vacuolar acidification, 25 nM BAF or an equal volume of DMSO were added to the macrophage cells 30 min prior to infection and maintained throughout the infection process.

### Wide-Field Fluorescence and Confocal Microscopy

All the wide-field images were collected using an Olympus IX71 Inverted Microscope (Applied Precision DeltaVision Deconvolution microscope system) equipped with 100X, 1.4 numerical aperture (NA) objective lens with a mercury arc bulb (OLYMPUS U-RFL-T: Mercury 150W) as the illumination source. Image capture was performed with the CoolSnap HQ, a fast, high resolution, high quantum efficiency, cooled CCD camera (Photometrics CoolSNAP HQ2 (CCD); 1,392 x 1,040 pixels; 11 fps). Two sets of images were taken corresponding to (i) donor emission wavelength (525/36 nm) upon donor excitation (490 ± 20 nm) (donor image: D), and (ii) acceptor emission wavelength (666 nm long pass) (acceptor FRET: A) upon donor excitation (490/20 nm). Acceptor images (I) acceptor emission wavelength (666 nm long pass) upon acceptor excitation (645 ± 20 nm) were obtained for cells clamped at pH 5.2 and 7.1. Confocal imaging was carried out on an inverted Nikon A1Rsi-Polarizing Module equipped with CFI Plan ApochromatVC 100X, 1.4 numerical aperture (NA) objective lens by using a Coherent CUBE laser at 488 excitation with set appropriate dichroics. Excitation at 470/40 nm wavelength was performed through a 510 nm dichroic mirror, and emission was recorded through a 525/50 nm band pass filter on a Andor DU897 EMCCD camera to yield the donor image (D). Similarly, emission was recorded through a 700/75 nm band pass filter upon similar excitation to generate the FRET intensity (A). Multiple Z sections were taken (0.5 μm apart) and each image is represented as sum slices. Images were analyzed using ImageJ ver.1.42. All of the images were background-subtracted by taking the mean intensity over an adjacent cell-free area. Autofluorescence of unlabeled *Salmonella* in RAW 264.7 cells was measured and used to correct the determined pH values. However, there was no difference in the intracellular pH values when we corrected for auto-fluorescence compared with the uncorrected values. Because the measurement is ratiometric, auto-fluorescence did not influence the determined pH values. Images showing the D/A values were obtained by using the ratio plus plugin of ImageJ software. The high and low values were color-coded and calibrated to their respective pH values as described in the main text. For all studies in macrophages, the mean D/A intensity of at least thirty bacterial cells was measured and is represented as the mean ± SEM of three independent batches of experiments.

### Measurement of BCECF in *Salmonella*

The free acid form of BCECF was suspended in buffers ranging from pH 5.0 to 7.2. Fluorescence intensities were measured at excitation wavelengths of 488 and 440 nm and the emission wavelength was recorded at 525 nm in a Tecan spectrophotometer (GENios). This generated the in vitro standard curve. Overnight cultures of WT and the *ompR* null mutant of *Salmonella* were clamped in 100 mM potassium phosphate buffer at various pH containing 40 μM nigericin and incubated in the presence of 20 μM BCECF-AM. Paired images were obtained by using excitation wavelengths of either 440 nm (pH-insensitive wavelength) or 488 nm (pH-sensitive wavelength). Fluorescence emission was recorded through a 525/50-nm band pass filter on a Andor DU897 EMCCD camera on an inverted Nikon A1Rsi-Polarizing Module by using Coherent CUBE lasers at 488 and 440 excitation with set appropriate dichroics. The ratios were plotted as a function of pH, and are comparable to the in vitro calibration curve. WT and the *ompR* null mutant of *Salmonella* were incubated at either acidic pHe (5.6) or neutral pHe (7.2) for 2 h. 20 μM BCECF-AM was added to the cultures for 30 min before imaging. To determine whether acidification was growth phase dependent, cells were grown until O.D. = 0.56 (log phase) or O.D. ≈ 2.3 (stationary phase) before the addition of the probe. The ratios of the fluorescence intensities of emission channel (525 nm) upon 488 nm excitation and 440 nm excitation were obtained as before. Cells were placed on microscope slides (Marienfeld Germany) coated with 1% agarose and then imaged. Images were first corrected for background fluorescence by subtracting an image of a cell-free area and analyzed by Image J imaging software.

### Preparation of *Salmonella* Spheroplasts

I-switch single labeled with Alexa 647 (I_A647_) was transformed in *Salmonella* as described above. Spheroplasts were prepared as described in [61] with the following modifications; Cells were resuspended after recovery in cold hypertonic solution A (0.75M sucrose 10 mM TrisHCl pH 7.8). 5 mg/ml of lysozyme solution was gently added to the cell suspension and the mixture was kept on ice for 5 min. EDTA solution (1.5 mM EDTA pH 7.5) was then added slowly over 10 min. Cells were centrifuged at 4,000 rpm for 20 min and re-suspended in solution D (0.25M sucrose 3 mM Tris-HCl pH 7.8, 1 mM EDTA pH 7.5). Cells were imaged on Applied Precision DeltaVision Deconvolution microscope system.

### Construction of Mutants and HA/GFP/mCherry Tagged Strains

The *sseJ*-HA fusion or *ompR* DNA sequences were amplified using primers XhoI-PsseJ #F and sseJ-HA-BamHI #R or KpnI-PompR #F and envZ*-*HindIII #R from *Salmonella* 14028s genomic DNA. The PCR products and low copy promoter-less pWSK29 vector were double-digested with restriction enzymes XhoI and BamHI or KpnI and HindIII (Thermo Fisher Scientific), and the digested samples were ligated with the Rapid DNA Ligation Kit (Thermo Fisher Scientific). The *cadA*, *cadB*, *cadC*, *ompC*, *ompF*, *mgtC*, *atpB*, and *sseB* genes were deleted from the *Salmonella* chromosome using λ-Red recombination techniques as described previously [62,63]. A *cadBA* over-expressed strain was generated by cloning *cadBA* into plasmid pBR322, replacing the *bla* gene with *cadBA*. SsaC-GFP and SsaJ-mCherry were constructed as follows: plasmid pWSK29 containing *ssaC* with the *spiC* promoter fused before GFP and *ssaJ* with the *sseA* promoter fused before mCherry were inserted in *ssaC* and *ssaJ* null strains, respectively. Primers are listed in S1 Table. A list of strains and plasmids are provided in S2 Table.

### Immunofluorescence Detection in Macrophages

For every time point post-infection, cells were fixed with 3% paraformaldehyde for 10 min at RT followed by three PBS washes. Cells were probed with various primary and secondary antibodies in PBS containing 2% BSA and 0.1% saponin. Antibody staining was done sequentially for 1 h, and after each incubation, washes were performed five times with PBS. The HA epitope tag was detected with the monoclonal HA antibody at 1:500 (Covance); LAMP-1 was detected with fluorescein isothiocyanate (FITC)-conjugated rabbit monoclonal antibody at 1:500 (Research Diagnostics); *Salmonella* was detected with mouse polyclonal anti-lipopolysaccharide (anti-LPS) antibodies at 1:500 (Difco). Secondary antibody detection was performed with various anti-rabbit or anti-mouse antibodies conjugated to Alexa-488 and cy5.

### Determination of Intracellular *Salmonella* Replication in Macrophages

To determine intracellular replication within macrophages, a similar protocol was followed as reported in [64] with slight modification. *Salmonella* was electroporated with non-labeled I-switch, and used for infection (see text for details). At 2 and 16 h post-infection, cells were washed repeatedly in PBS and lysed in 0.1% Triton-X100 at RT for 15min. Samples were then serially diluted and plated on LB to enumerate the intracellular bacteria. Replication was described as the CFU/ml counted and represented as an average of three plates for all time points.

### Electrophoretic Mobility Shift Assay

Electrophoretic mobility shift assays (EMSAs) were performed using the Lightshift chemi-luminescence EMSA kit (Pierce) according to the manufacturer’s instructions described in [65]. The upstream regions of *cadC* (354 bp), *cadB* (361 bp), and *cadA* (264 bp upstream to 34 bp downstream) were amplified using biotinylated oligonucleotides. Ten fmol of biotinylated DNA was used in a 15 μl reaction containing binding buffer (10 mM Tris, pH 7.5, 50 mM KCl) along with 2.5% glycerol, 5 mM MgCl_2_, 0.05% Nonidet P-40, and 1μg poly(dI-dC)). OmpR or OmpR~P protein was added at the concentrations indicated, and samples were separated by electrophoresis on 5% non-denaturing acrylamide gels run in 0.5X Tris-acetate buffer with EDTA. Following electrophoresis, DNA was electro-transferred to a nylon membrane and detected using the biotin detection system (Pierce).

### RNA Isolation and Quantitative Real-Time RT-PCR

*Salmonella* strains were grown in SPI-2 inducing Minimal Magnesium Medium (pH 5.6) as described previously [60] to O.D ~0.6. The total RNA was isolated using an RNeasy mini kit (Qiagen). After DNase treatment of the isolated RNA, cDNA was synthesized using the iScript Reverse Transcription Supermix (Biorad). Quantification of cDNA was carried out using SsoFast ^TM^ Eva Green Supermix (Bio-Rad), and real-time amplification of the PCR products was performed using the iCycler iQ real-time detection system (Bio-Rad). The mRNA expression level of the target gene was normalized relative to the 16S rRNA expression level.

### Acid Tolerance Response Assay

Cultures were grown in LPM media (pH 7.0) to O.D ~1.5 as described in [66] with slight modifications. Unadapted cultures were immediately shifted to pH 3.0 acidified by HCl. Adapted cultures were shifted to pH 4.5 for 2 h prior to the challenge at pH 3.0. Viable cells were calculated by plating aliquots of serially diluted cultures by standard plate count.

### Measurement of Intracellular ATP in *Salmonella*

Intracellular ATP levels using Tecan spectrophotometer as described with modifications [37]: Overnight cultures of *Salmonella* in LB were sub-cultured in MgM (7.2) for 24 h. The cultures were washed in MgM (5.6) and inoculated in 5 ml of the same media for 5 h. Cells were normalized by the OD_600_ and re-suspended in 500 μl of (PBS). Nucleic acids were extracted by adding ice cold 1% Trichloroacetic acid (TCA) and 2 mM EDTA for 15 min. The extracts were neutralized with 100 μl of neutralization buffer and centrifuged for 15 min. The supernatant was diluted 2-fold with L buffer and the luciferase reaction was initiated using an ATP determination kit (Invitrogen) according to the manufacturer's instructions. Intracellular ATP levels (μM) were converted using reference to standards of known concentration.

### Immunoblotting

Overnight cultures of *Salmonella* WT and *ssaC* null mutant strains in LB were sub-cultured in MgM (7.2) for 24 h. The cultures were washed either in MgM (5.6) or MgM 7.2 and inoculated in 50 ml of the same media for 5 h. For isolation of the secreted protein, cells were removed from the culture by centrifugation (5,500 x *g*, 20 min, 4°C), and the supernatant was filtered through a 0.22 μm pore size binding filter (Millipore, Billerica, MA). The secreted fraction was isolated by 10% trichloroacetic acid precipitation, and the protein pellet was washed three times with −20°C acetone and then air dried. Equivalent amounts of cellular protein, adjusted according to the optical densities, were used for the whole cell fraction. The protein samples were separated by 12% SDS-PAGE and transferred to PVDF membrane (Millipore). The membrane was incubated with anti-SseB (1:10,000) or anti-GroEL (1:5,000) antibodies in PBS buffer (with 0.05% Tween 20 and 1% Skim Milk) followed by anti-rabbit secondary antibody (1:5,000, Santa Cruz Biotechnology).

## Supporting Information

S1 Data All the numerical data. (XLSX)

S1 Fig In vitro calibration curve and intracellular localization of the I-switch in the *Salmonella* cytoplasm. **(A)** An in vitro calibration profile was obtained with the dual labeled (Alexa-488/647) I-switch along with the donor only and acceptor only (see text for details). The mean donor intensity (D) at 520 nm and mean acceptor intensity (A) at 666 nm were recorded. D/A values at each pH were plotted as a function of pH to generate the in vitro calibration curve. Cross talk (0.03%) (acceptor emission at the donor excitation wavelength) and bleed-through (0.3%) (donor emission at the acceptor excitation wavelength) were measured with acceptor-only and donor-only species. **(B)** Spheroplasts were prepared with *Salmonella* electroporated with I_A647_. Spheroplasts exhibit a characteristic spherical shape due to removal of the cell wall. Representative epifluorescence images of acceptor emission wavelength (666 nm) upon acceptor excitation (645/20 nm) were obtained. Fewer spheroplasts were obtained from cells electroporated with the I-switch, presumably as a result of increased cell wall rigidity after recovery from electroporation. However, it can be clearly seen that I_A647_ is inside the spheroplasts, confirming the I-switch localization in the cytoplasm. Scale bar, 3 μm.
(TIF)

S2 Fig OmpR regulates the acid tolerance response. **(A)** Cultures of WT, *ompR* null and *ompR* null mutant complemented with *ompR* supplied in *trans* were grown in LPM media as described in Materials and Methods. Unadapted cells were immediately shifted to pH 3.0 for 2 h, whereas adapted cultures were shifted to an intermediate pH 4.5 for 2 h prior to acid shock at pH 3.0. Viable counts immediately after the acid challenge to pH 3.0 were considered as 100%. Error bars represent the mean ± SD (*n* = 3).
(TIF)

S3 Fig The *Salmonella* cytoplasm is acidified upon acid stress independently of the growth phase. **(A)** A WT culture was incubated in MgM (5.6) until O.D. = 0.56 (log phase) or O.D. ≈ 2.3 (stationary phase). Twenty μM BCECF-AM was added 30 min before imaging. Representative epifluorescence of the emission intensity detected at 525 nm when excited at 488 nm/440 nm were obtained at the indicated time points. Scale bar, 3 μm. **(B)** The plot indicates the intracellular pH of the log phase or stationary phase cultures of WT *Salmonella* at the indicated time points. Error bars represent the mean ± SEM (*n* = 3).
(TIF)

S4 Fig The intracellular pH probe BCECF-AM reports similar *Salmonella* acidification in response to extracellular acid stress compared to the I-switch. **(A)** The free acid form of BCECF was used to obtain the in vitro data points over a range of pH values. Overnight cultures of WT and *ompR* null mutant of *Salmonella* were clamped in 100 mM potassium phosphate buffer at various pH containing 40 μM nigericin and incubated in the presence of 20 μM BCECF-AM. The ratios of the fluorescence intensities of emission channel (525 nm) upon 488 nm excitation and 440 nm excitation were obtained using the Nikon A1R confocal microscope. The ratios were plotted as a function of pH, and show perfect overlap with the in vitro calibration curve, corroborating the intracellular location of the I-switch. **(B)** Representative pseudo-colored images of emission intensity detected at 525 nm when excited at 488 nm versus 440 nm were obtained with cells incubated for 120 min at either acidic pH or neutral pH. Using ImageJ software, the ratio images were then color-coded with blue (Ratio = 0.1) to red (Ratio = 1). Thirty cells were counted to determine the intracellular pH of *Salmonella*. Scale bar, 3 μm. **(C)** A comparison of results obtained from the I-switch and BCECF-AM to determine the intracellular pH of *Salmonella* after 120 min in either acidic or neutral pH.
(TIF)

S5 Fig *Salmonella* recovered from the SCV exhibit rapid cytoplasmic acidification. **(A)** RAW macrophages were infected with (I_A488/A647_)-incorporated WT *Salmonella* for 3 h as described in Materials and Methods. At the designated time intervals, macrophages were washed with PBS and lysed with 0.1% Triton X-100 for 20 min at RT. The enumerated bacteria from macrophages were mounted on a clean slide containing a 1% agarose pad and imaged on Applied Delta Vision wide field fluorescence microscope. The D/A values were obtained from at least 30 cells at the indicated time points. Each experiment was performed in triplicate and representative images are shown. Scale bar, 3μm. **(B)** Intracellular pH was plotted from the D/A ratio obtained at each analyzed time point. Error bars represented as the mean ± SEM.
(TIF)

S6 Fig The *Salmonella* cytoplasm rapidly follows the vacuolar pH. RAW264.7 macrophages were pre-treated with 25 nM BAF for 30 min prior to infection. Macrophages were infected with (I_A488/A647_)_-_electroporated *Salmonella* in the presence of BAF for 1 h. The cells were washed three times with PBS and no BAF was added for the next 2 h (arrow). The intracellular pH of *Salmonella* decreased upon removal of BAF from the media as the vacuolar pH acidified. **(A)** Representative images of D/A ratios are shown at various times post-infection. Scale bar, 3 μm. **(B)** The D/A ratio of ~30 cells were calculated at indicated time points of infection. Error bars represented as the mean ± SEM (*n* = 3).
(TIF)

S7 Fig Known OmpR targets are not required for intracellular acidification. **(A)** IA488/IA647-incorporated *ssaC*, *ompC*, *ompC*/*ompF* and Δ*mgtC* null mutants of *Salmonella* were incubated in either acidic pHe (5.6) or neutral pHe (7.2) for the indicated times. Representative epifluorescence of the D/A ratio images are shown for the Δ*ssaC*, Δ*ompC*, Δ*ompC*/*ompF* and Δ*mgtC* strains. Scale bar, 3 μm. **(B)** The plot indicates the intracellular pH of the Δ*ssaC*, Δ*ompC*, Δ*ompC*/*ompF* and Δ*mgtC* mutants at pHe 5.6 and pHe 7.2, compared to WT at pHe 5.6 and pHe 7.2. The D/A ratios of 50 cells were analyzed at each time point and the pH values were determined from the intracellular standard curve. Similar results were obtained for three independent experiments. Error bars were removed for clarity.
(TIF)

S8 Fig Over-expression of *cadBA* completely over-rides OmpR-dependent acidification. **(A)** *Salmonella* cultures of I_A488_/I_A647-_incorporated WT, *cadBA* null mutant and *cadBA* over-expressed strain (*cadBA* is under the control of *bla* promoter) were incubated at either acidic pH_e_ (5.6) or neutral pH_e_ (7.2) at indicated time points. Representative epifluorescence of the D/A ratio images are shown for WT, the *cadBA* mutant and the *cadBA* over-expressed strains of *Salmonella*. Scale bar, 3 μm. **(B)** A plot of the intracellular pH of *Salmonella* WT, *cadBA* null mutant and *cadBA* over-expressed strain at pH_e_ 5.6 and pH_e_ 7.2 over time. The D/A ratios of 50 cells were analyzed at each time point and the pH values were determined from the intracellular standard curve. Error bars represent the mean ± SEM (*n* = 3).
(TIF)

S9 Fig OmpR up-regulates *atpB*, resulting in increased intracellular ATP levels. **(A)** Intracellular ATP levels were determined for the WT, *ompR* null, the *ompR* null mutant complemented with *ompR* supplied in *trans*, an *mgtC* null and *atpB* null strains grown in MgM (pH 5.6), as described in Materials and Methods. 5 μM of protonophore CCCP was used as a control. Error bars represent the mean ± SEM (*n* = 3). **(B)** mRNA levels of *atpB* and *mgtC* genes were determined by qRT-PCR from WT and *ompR* null strains as described in Materials and Methods. The error bars represent the mean ± SD (*n* = 3).
(TIF)

S10 Fig Polar localization of the SPI-2 needle complex. **(A)** Representative bright field, fluorescence and merged images of *Salmonella* harboring SsaC-GFP (green) and SsaJ-mCherry (red) incubated at pH_e_ 5.8 for 7 h and imaged on an Applied Delta Vision wide field fluorescence microscope. For SseB localization, WT *Salmonella* was immunostained for SseB (green) followed by Alexa-488 secondary antibody. Scale bar, 2μm. (B) Immunoblot analysis of whole cell and secreted protein fractions prepared from WT and an *ssaC* null mutant of *Salmonella* as described in Materials and Methods. Anti-GroEL antibodies were used as loading controls.
(TIF)

S11 Fig Translocon complex is required for effector secretion. RAW264.7 macrophages were infected with *Salmonella sseB* null strain harboring p*sseJ-HA* for expression of SseJ-HA. Cells were immunostained for *Salmonella* LPS (green) and HA epitope (red) followed by Alexa488- or Alexa647-labeled secondary antibodies, respectively, for various time points as indicated. Samples were imaged by confocal microscopy and analyzed using ImageJ software. Scale bar, 3 μm. No SseJ secretion was observed in the *sseB* null strain of *Salmonella*.
(TIF)

S12 Fig Dilution of I-switch positive *Salmonella* cells upon cell division. **(A)** Single labeled I-switch (I_A647_) incorporated WT *Salmonella* was incubated in MgM pH 5.6 for 2.5 h. Representative merged images of phase contrast and epifluorescence of the acceptor channel (green) were obtained on an Applied Delta Vision wide field fluorescence microscope every 30 min. Scale bar, 3μm. **(B)** The percentage of I-switch positive cells is plotted over time. After 2 h of incubation, approximately 20% cells contained visible I-switch.
(TIF)

S13 Fig The *ompR* mutant strain and the *cadBA* over-expressed strain were defective for replication within RAW macrophages. Macrophages were seeded at a density of 10^5^ cells per well in 24-well tissue culture plates, 24 h before use. WT, *ompR* null, *ssaC* null and *cadBA* over-expressed strains of *Salmonella* were added as described in Materials and Methods. At 2 and 16 h post-infection, host cells were lysed with 0.1% Triton X-100 for 10 min and cultured for enumeration of intracellular bacteria (gentamicin-protected) on to LB agar. All infections were performed in triplicate. The results are represented as the mean ± SEM.
(TIF)

S14 Fig *Salmonella* is intracellular during macrophage infection and co-localizes with the vacuolar marker LAMP-1. **(A)** LAMP-1 localizes to the SCV. To determine that *Salmonella* containing the I-switch were localized to the SCV in macrophages, immuno-labeling was performed with unlabeled I-switch electroporated *Salmonella* stained with LPS (green) and LAMP-1 (red). The merged image indicates that *Salmonella* containing the I-switch were localized primarily in the SCVs. Images were obtained using the Nikon A1R confocal microscope. Localization resulted in yellow staining of *Salmonella* cells as analyzed by Image J software. Scale bar = 3 μm. **(B)** Results of three independent experiments in which SCVs stained with LAMP-1 were scored for WT *Salmonella* and an *ompC* mutant at 0.5, 4, and 6 h post-infection. Co-localization analysis was performed using ImageJ software. The values represent the mean ± SEM.
(TIF)

S1 Table Sequences of primers used in this study. (DOCX)

S2 Table Strains and plasmid vectors used in this study. (DOCX)

## Abbreviations

A: acceptor fluorescence intensity
BAF: bafilomycin A1
BCECF-AM: 2',7'-Bis-(2-Carboxyethyl)-5-(and-6)-Carboxyfluorescein, Acetoxymethyl Ester
CCCP: carbonyl cyanide m-chlorophenyl hydrazone
D: donor fluorescence intensity
EMSA: Electrophoretic mobility shift assays
FRET: fluorescence resonance energy transfer
pH_i_: intracellular pH
pH_e_: extracellular pH
qRT-PCR: quantitative real-time polymerase chain reaction
RT: room temperature
SCV: *Salmonella* containing vacuole
Sifs: *Salmonella*-induced filaments
SPI: *Salmonella* pathogenicity island
T3SS: type III secretion systems
WT: wild type
