# Supplementary figures and images for: A FRET-Based DNA Biosensor Tracks OmpR-Dependent Acidification of Salmonella during Macrophage Infection
Source: PLoS Biol. 2015 Apr 14;13(4):e1002116. doi: 10.1371/journal.pbio.1002116 (PMC4397060; doi:10.1371/journal.pbio.1002116)

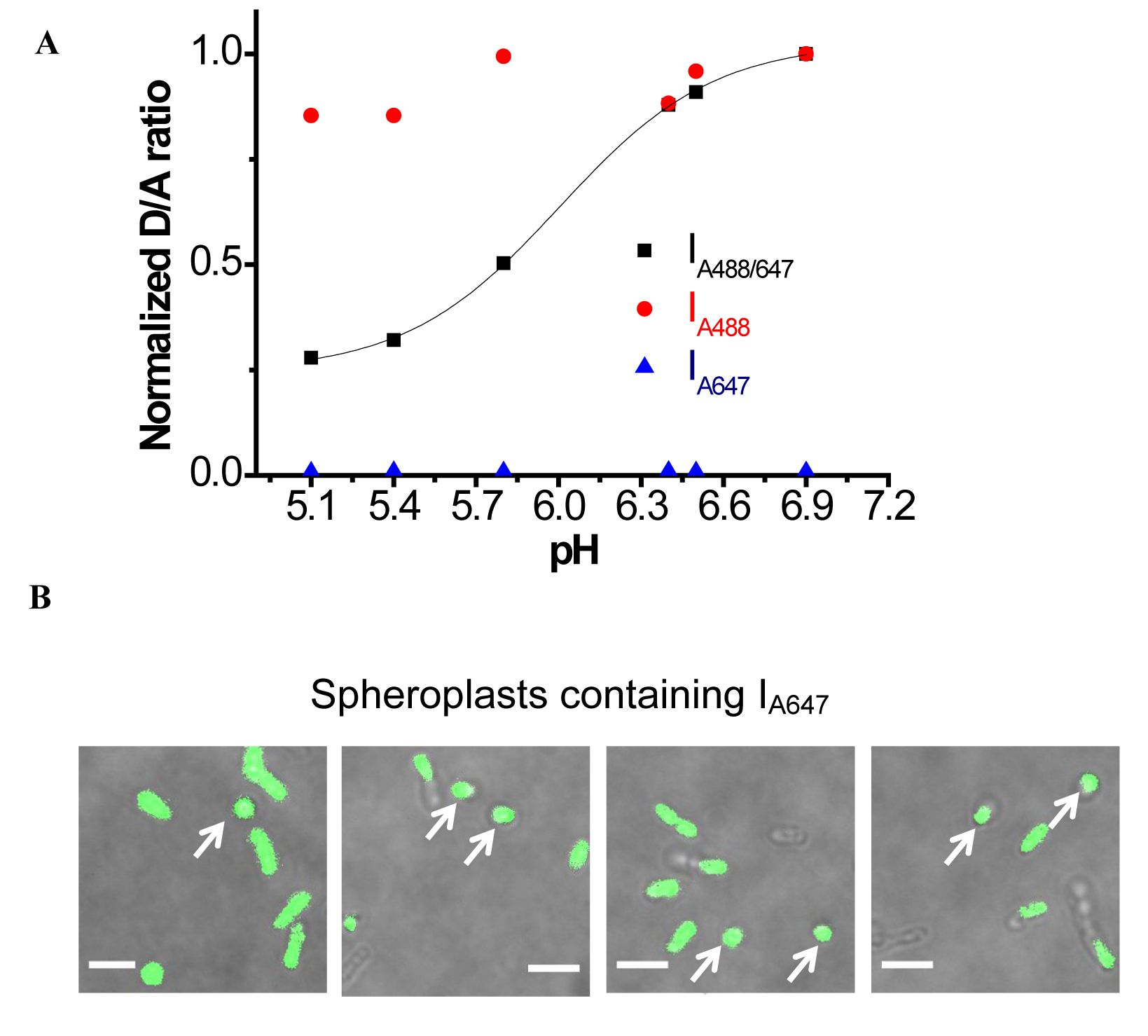

Supplement: S1 Fig — (A) An in vitro calibration profile was obtained with the dual labeled (Alexa-488/647) I-switch along with the donor only and acceptor only (see text for details). The mean donor intensity (D) at 520 nm and mean acceptor intensity (A) at 666 nm were recorded. D/A values at each pH were plotted as a function of pH to generate the in vitro calibration curve. Cross talk (0.03%) (acceptor emission at the donor excitation wavelength) and bleed-through (0.3%) (donor emission at the acceptor excitation wavelength) were measured with acceptor-only and donor-only species. (B) Spheroplasts were prepared with Salmonella electroporated with IA647. Spheroplasts exhibit a characteristic spherical shape due to removal of the cell wall. Representative epifluorescence images of acceptor emission wavelength (666 nm) upon acceptor excitation (645/20 nm) were obtained. Fewer spheroplasts were obtained from cells electroporated with the I-switch, presumably as a result of increased cell wall rigidity after recovery from electroporation. However, it can be clearly seen that IA647 is inside the spheroplasts, confirming the I-switch localization in the cytoplasm. Scale bar, 3 μm. (TIF) [file pbio.1002116.s002.tif]

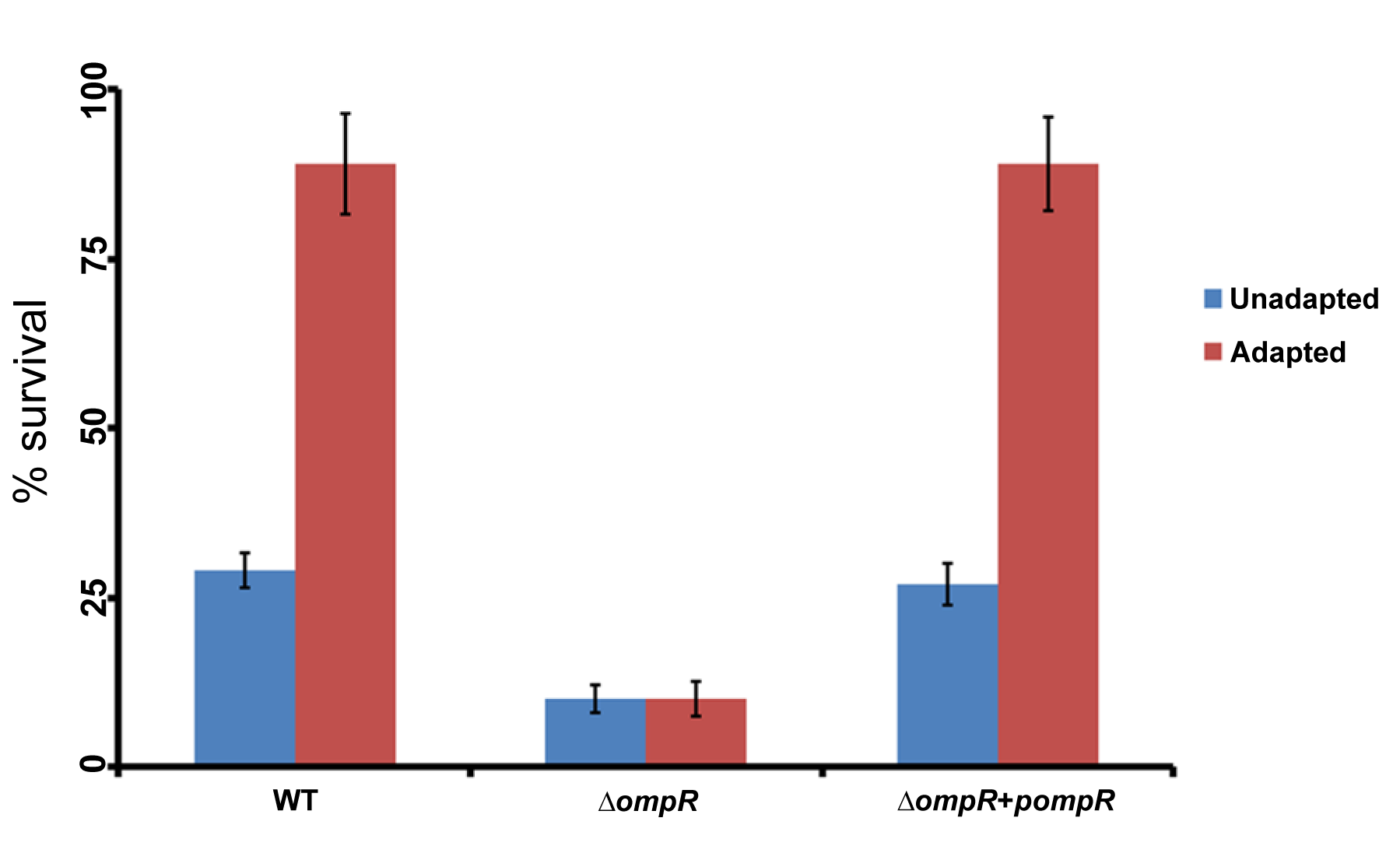

Supplement: S2 Fig — (A) Cultures of WT, ompR null and ompR null mutant complemented with ompR supplied in trans were grown in LPM media as described in Materials and Methods. Unadapted cells were immediately shifted to pH 3.0 for 2 h, whereas adapted cultures were shifted to an intermediate pH 4.5 for 2 h prior to acid shock at pH 3.0. Viable counts immediately after the acid challenge to pH 3.0 were considered as 100%. Error bars represent the mean ± SD (n = 3). (TIF) [file pbio.1002116.s003.tif]

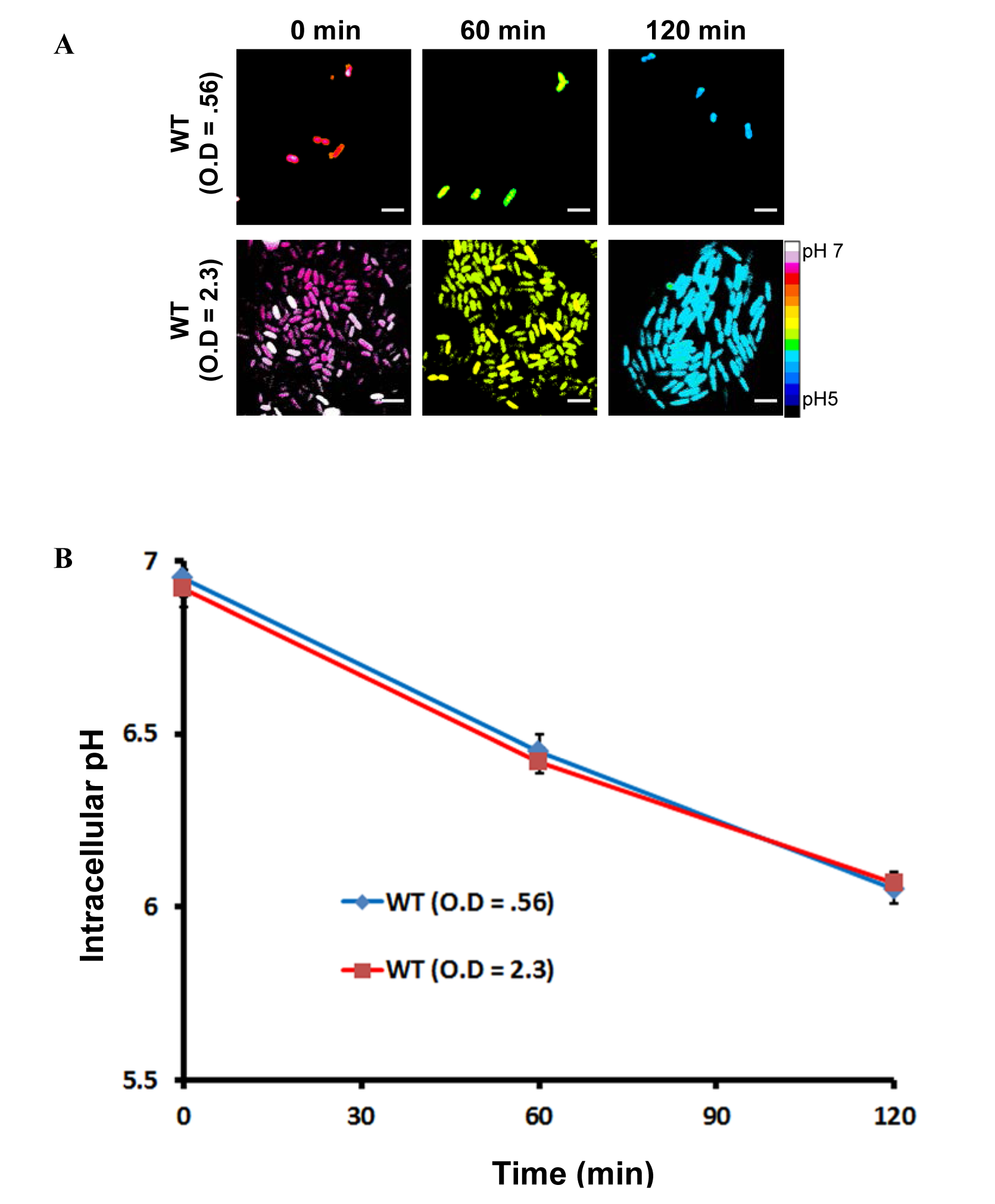

Supplement: S3 Fig — (A) A WT culture was incubated in MgM (5.6) until O.D. = 0.56 (log phase) or O.D. ≈ 2.3 (stationary phase). Twenty μM BCECF-AM was added 30 min before imaging. Representative epifluorescence of the emission intensity detected at 525 nm when excited at 488 nm/440 nm were obtained at the indicated time points. Scale bar, 3 μm. (B) The plot indicates the intracellular pH of the log phase or stationary phase cultures of WT Salmonella at the indicated time points. Error bars represent the mean ± SEM (n = 3). (TIF) [file pbio.1002116.s004.tif]

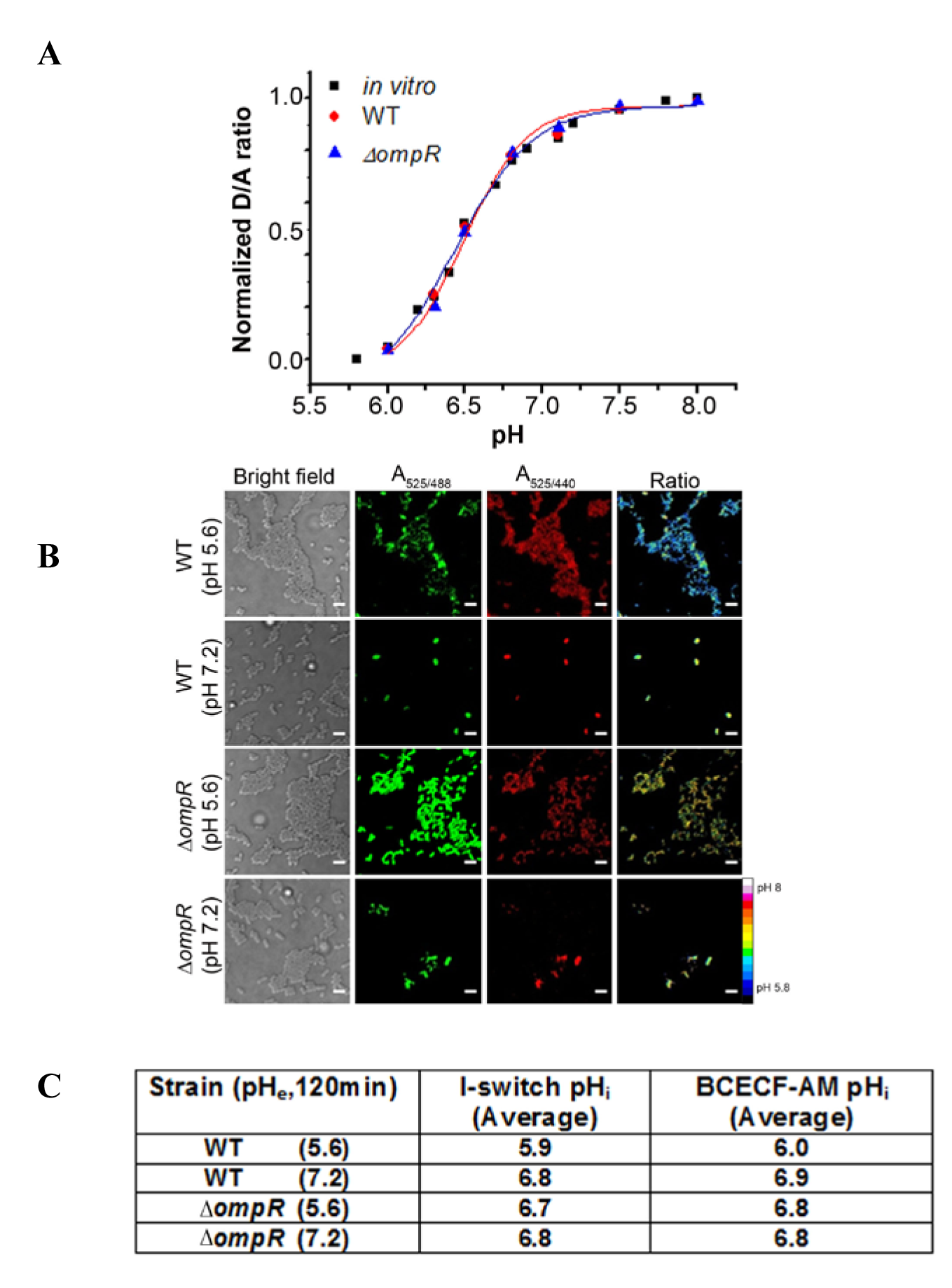

Supplement: S4 Fig — (A) The free acid form of BCECF was used to obtain the in vitro data points over a range of pH values. Overnight cultures of WT and ompR null mutant of Salmonella were clamped in 100 mM potassium phosphate buffer at various pH containing 40 μM nigericin and incubated in the presence of 20 μM BCECF-AM. The ratios of the fluorescence intensities of emission channel (525 nm) upon 488 nm excitation and 440 nm excitation were obtained using the Nikon A1R confocal microscope. The ratios were plotted as a function of pH, and show perfect overlap with the in vitro calibration curve, corroborating the intracellular location of the I-switch. (B) Representative pseudo-colored images of emission intensity detected at 525 nm when excited at 488 nm versus 440 nm were obtained with cells incubated for 120 min at either acidic pH or neutral pH. Using ImageJ software, the ratio images were then color-coded with blue (Ratio = 0.1) to red (Ratio = 1). Thirty cells were counted to determine the intracellular pH of Salmonella. Scale bar, 3 μm. (C) A comparison of results obtained from the I-switch and BCECF-AM to determine the intracellular pH of Salmonella after 120 min in either acidic or neutral pH. (TIF) [file pbio.1002116.s005.tif]

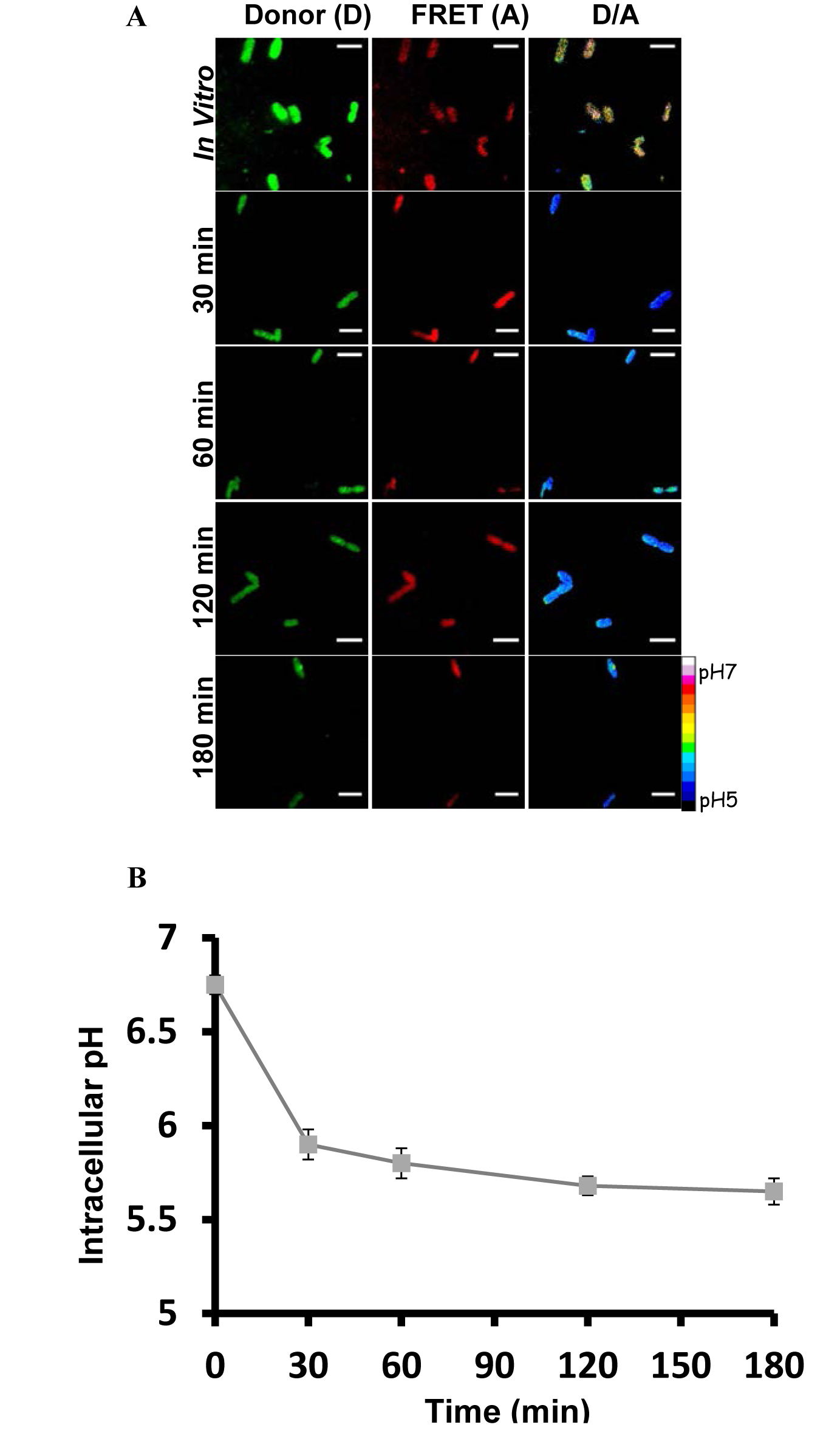

Supplement: S5 Fig — (A) RAW macrophages were infected with (IA488/A647)-incorporated WT Salmonella for 3 h as described in Materials and Methods. At the designated time intervals, macrophages were washed with PBS and lysed with 0.1% Triton X-100 for 20 min at RT. The enumerated bacteria from macrophages were mounted on a clean slide containing a 1% agarose pad and imaged on Applied Delta Vision wide field fluorescence microscope. The D/A values were obtained from at least 30 cells at the indicated time points. Each experiment was performed in triplicate and representative images are shown. Scale bar, 3μm. (B) Intracellular pH was plotted from the D/A ratio obtained at each analyzed time point. Error bars represented as the mean ± SEM. (TIF) [file pbio.1002116.s006.tif]

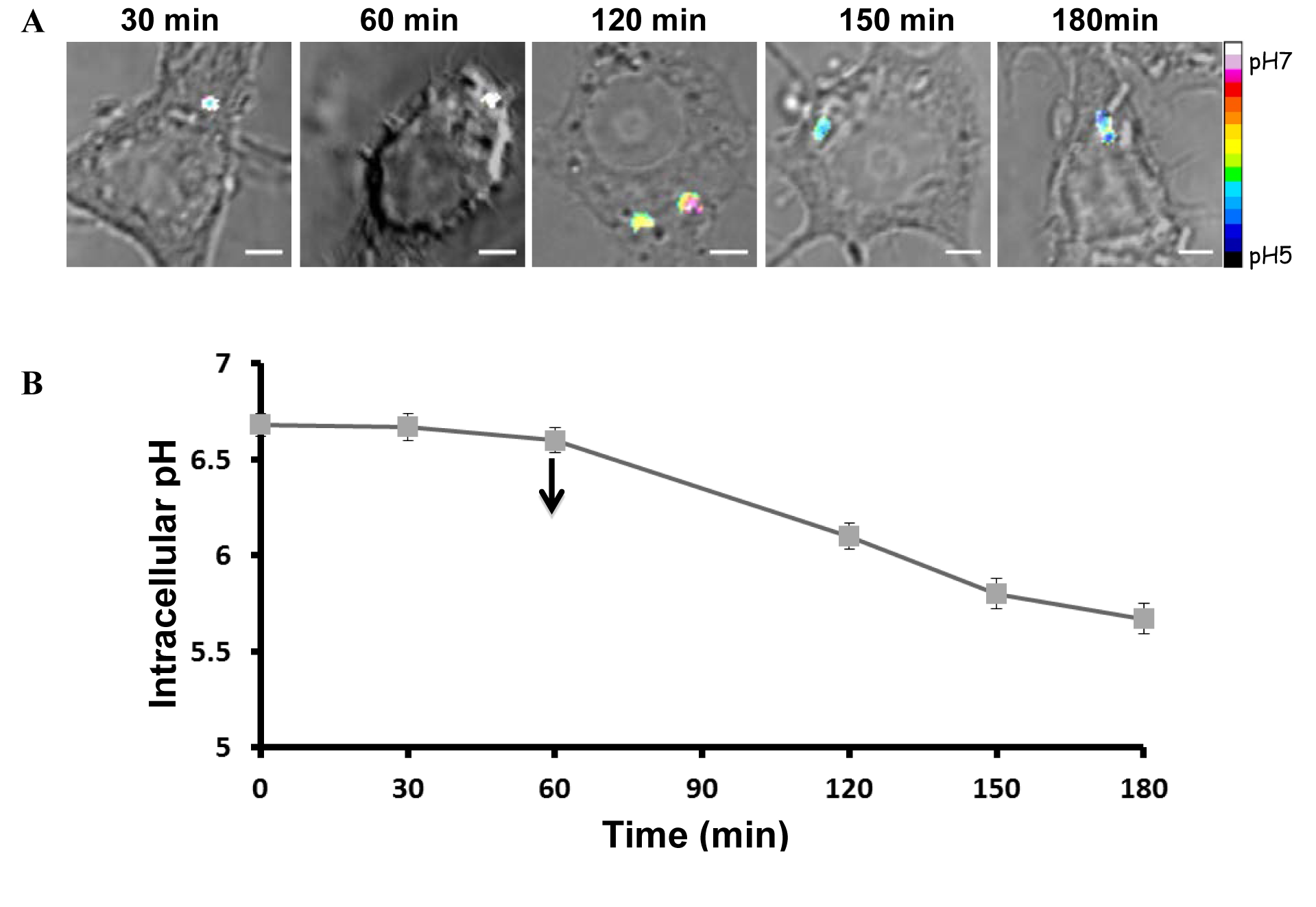

Supplement: S6 Fig — RAW264.7 macrophages were pre-treated with 25 nM BAF for 30 min prior to infection. Macrophages were infected with (IA488/A647)-electroporated Salmonella in the presence of BAF for 1 h. The cells were washed three times with PBS and no BAF was added for the next 2 h (arrow). The intracellular pH of Salmonella decreased upon removal of BAF from the media as the vacuolar pH acidified. (A) Representative images of D/A ratios are shown at various times post-infection. Scale bar, 3 μm. (B) The D/A ratio of ~30 cells were calculated at indicated time points of infection. Error bars represented as the mean ± SEM (n = 3). (TIF) [file pbio.1002116.s007.tif]

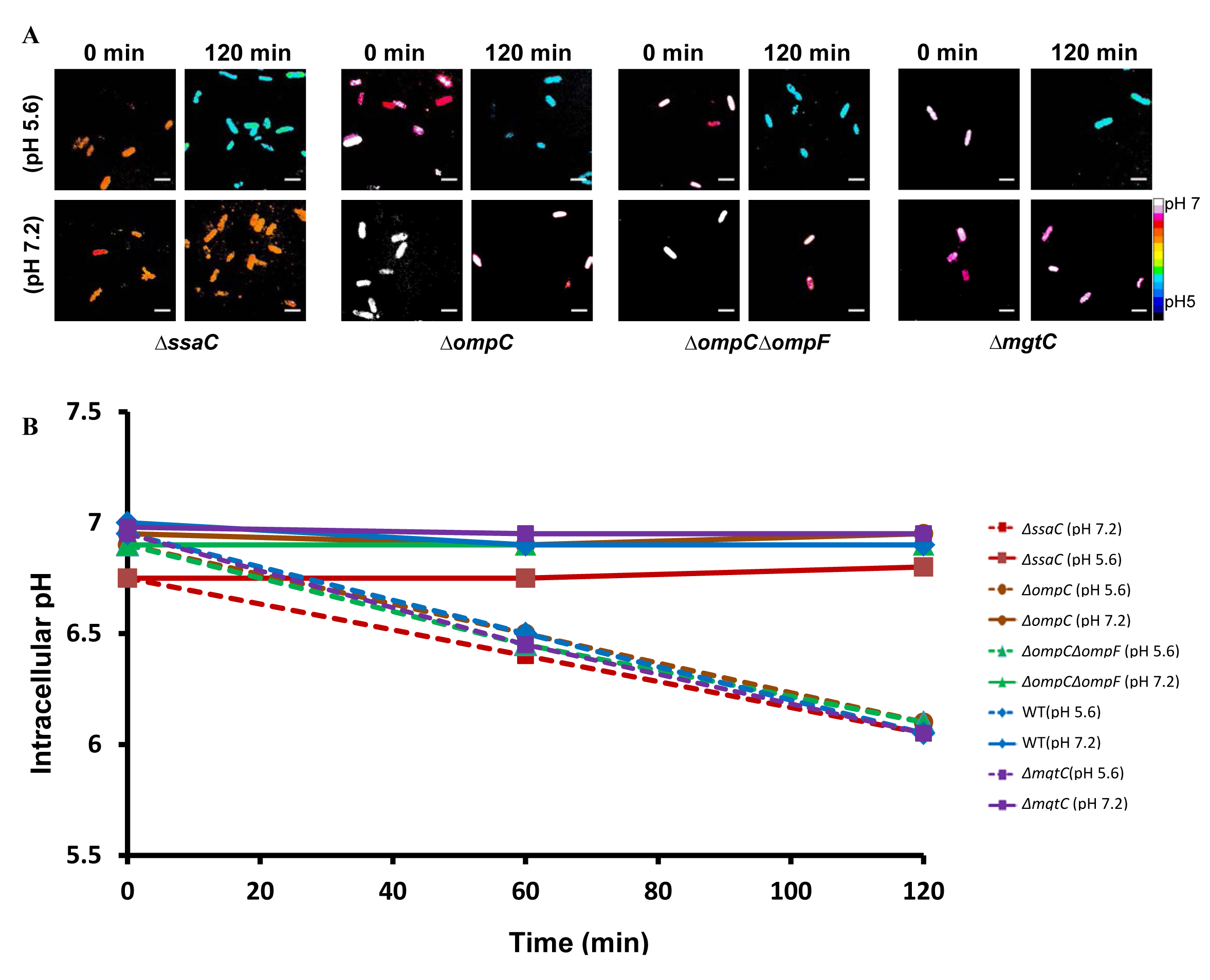

Supplement: S7 Fig — (A) IA488/IA647-incorporated ssaC, ompC, ompC/ompF and ΔmgtC null mutants of Salmonella were incubated in either acidic pHe (5.6) or neutral pHe (7.2) for the indicated times. Representative epifluorescence of the D/A ratio images are shown for the ΔssaC, ΔompC, ΔompC/ompF and ΔmgtC strains. Scale bar, 3 μm. (B) The plot indicates the intracellular pH of the ΔssaC, ΔompC, ΔompC/ompF and ΔmgtC mutants at pHe 5.6 and pHe 7.2, compared to WT at pHe 5.6 and pHe 7.2. The D/A ratios of 50 cells were analyzed at each time point and the pH values were determined from the intracellular standard curve. Similar results were obtained for three independent experiments. Error bars were removed for clarity. (TIF) [file pbio.1002116.s008.tif]

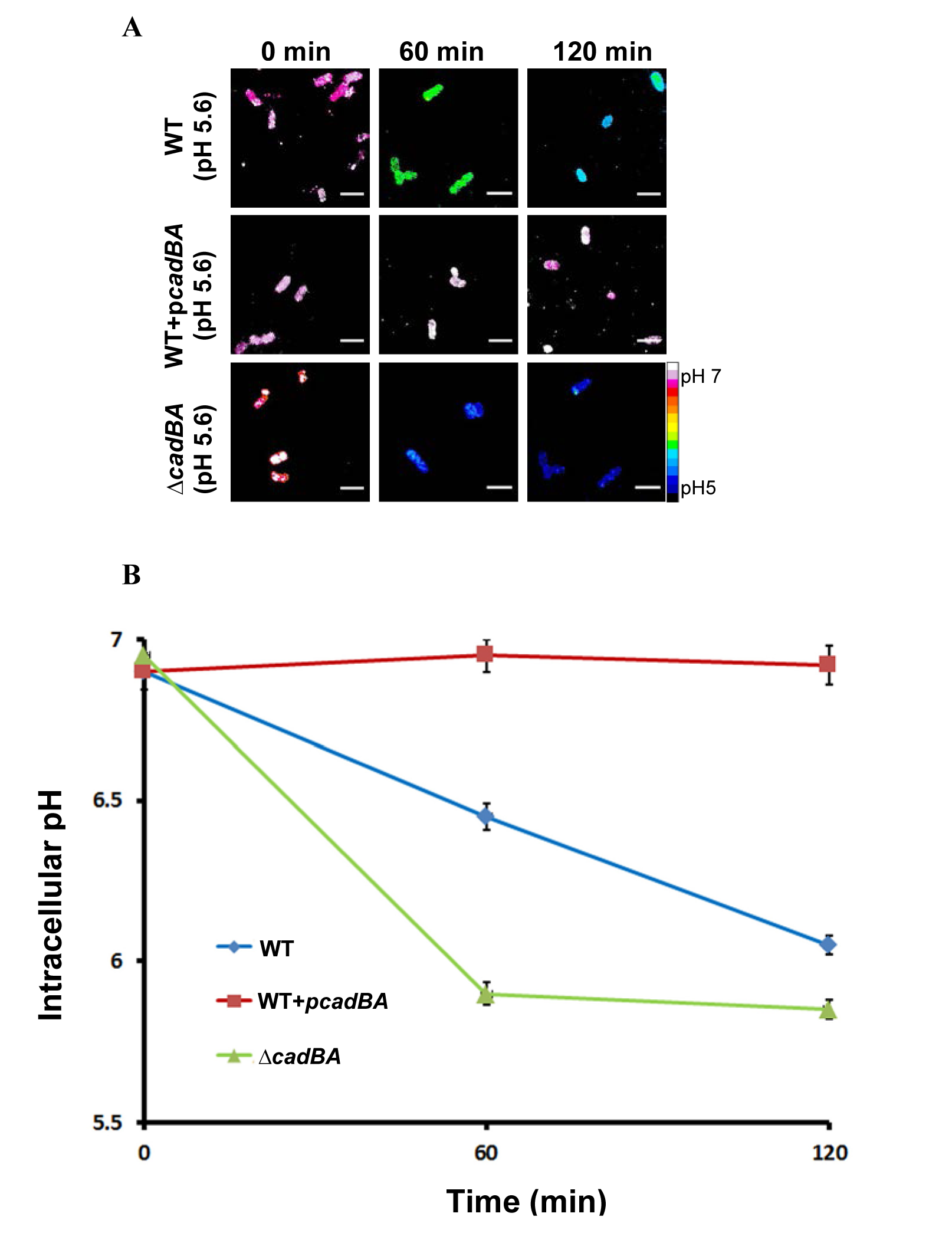

Supplement: S8 Fig — (A) Salmonella cultures of IA488/IA647-incorporated WT, cadBA null mutant and cadBA over-expressed strain (cadBA is under the control of bla promoter) were incubated at either acidic pHe (5.6) or neutral pHe (7.2) at indicated time points. Representative epifluorescence of the D/A ratio images are shown for WT, the cadBA mutant and the cadBA over-expressed strains of Salmonella. Scale bar, 3 μm. (B) A plot of the intracellular pH of Salmonella WT, cadBA null mutant and cadBA over-expressed strain at pHe 5.6 and pHe 7.2 over time. The D/A ratios of 50 cells were analyzed at each time point and the pH values were determined from the intracellular standard curve. Error bars represent the mean ± SEM (n = 3). (TIF) [file pbio.1002116.s009.tif]

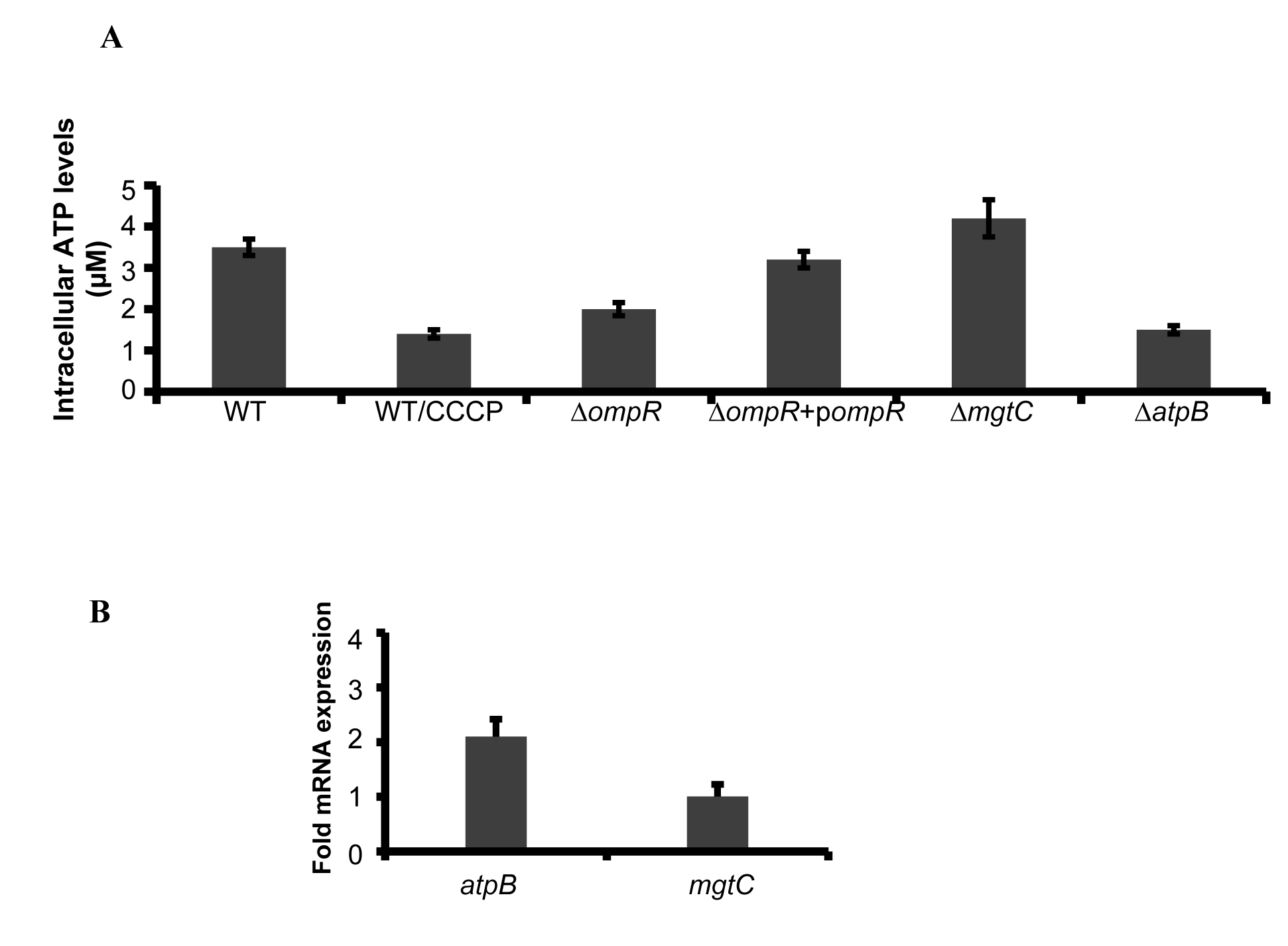

Supplement: S9 Fig — (A) Intracellular ATP levels were determined for the WT, ompR null, the ompR null mutant complemented with ompR supplied in trans, an mgtC null and atpB null strains grown in MgM (pH 5.6), as described in Materials and Methods. 5 μM of protonophore CCCP was used as a control. Error bars represent the mean ± SEM (n = 3). (B) mRNA levels of atpB and mgtC genes were determined by qRT-PCR from WT and ompR null strains as described in Materials and Methods. The error bars represent the mean ± SD (n = 3). (TIF) [file pbio.1002116.s010.tif]

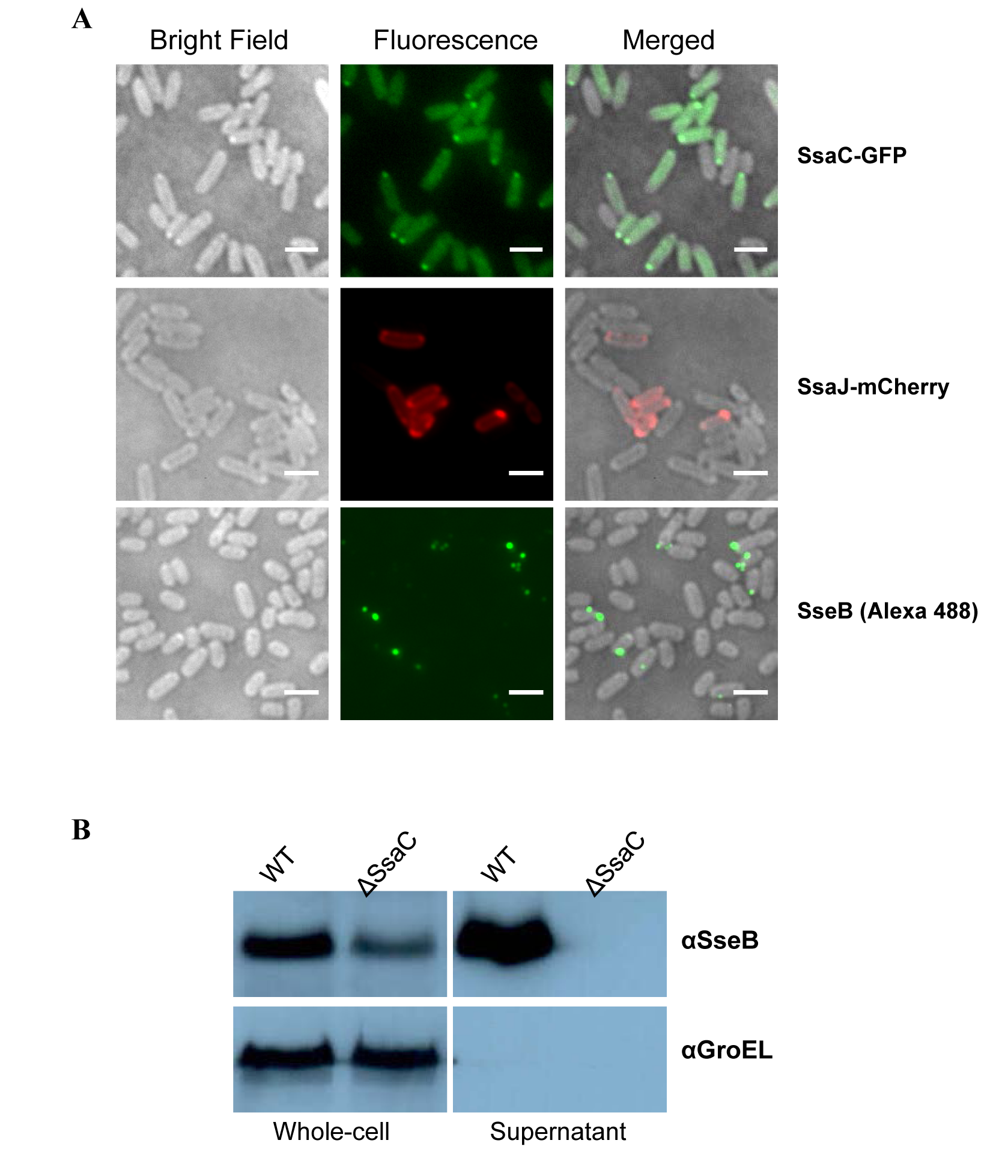

Supplement: S10 Fig — (A) Representative bright field, fluorescence and merged images of Salmonella harboring SsaC-GFP (green) and SsaJ-mCherry (red) incubated at pHe 5.8 for 7 h and imaged on an Applied Delta Vision wide field fluorescence microscope. For SseB localization, WT Salmonella was immunostained for SseB (green) followed by Alexa-488 secondary antibody. Scale bar, 2μm. (B) Immunoblot analysis of whole cell and secreted protein fractions prepared from WT and an ssaC null mutant of Salmonella as described in Materials and Methods. Anti-GroEL antibodies were used as loading controls. (TIF) [file pbio.1002116.s011.tif]

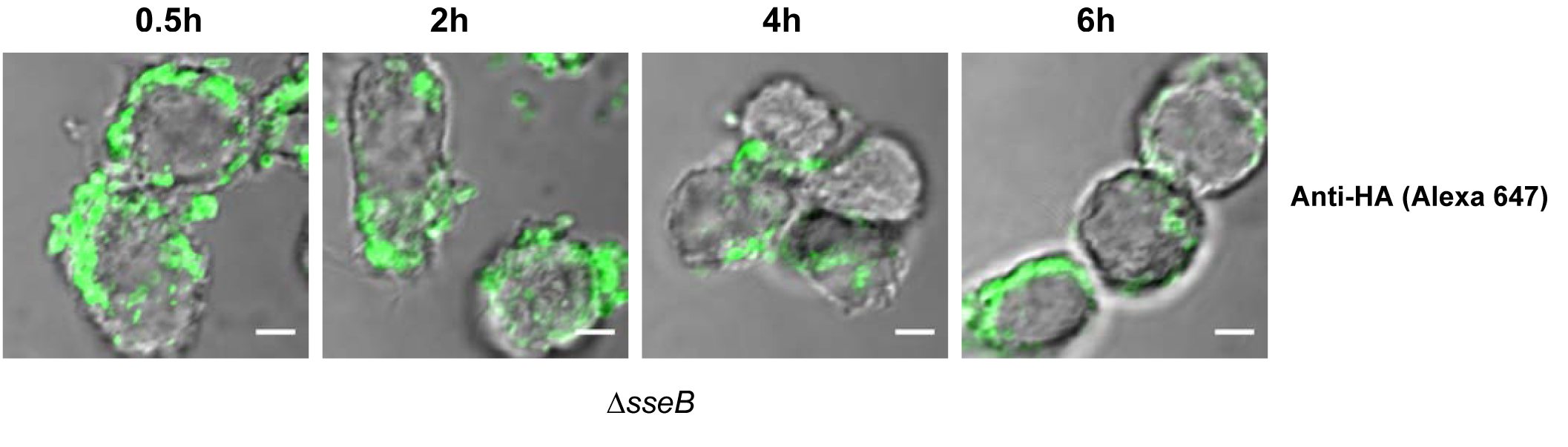

Supplement: S11 Fig — RAW264.7 macrophages were infected with Salmonella sseB null strain harboring psseJ-HA for expression of SseJ-HA. Cells were immunostained for Salmonella LPS (green) and HA epitope (red) followed by Alexa488- or Alexa647-labeled secondary antibodies, respectively, for various time points as indicated. Samples were imaged by confocal microscopy and analyzed using ImageJ software. Scale bar, 3 μm. No SseJ secretion was observed in the sseB null strain of Salmonella. (TIF) [file pbio.1002116.s012.tif]

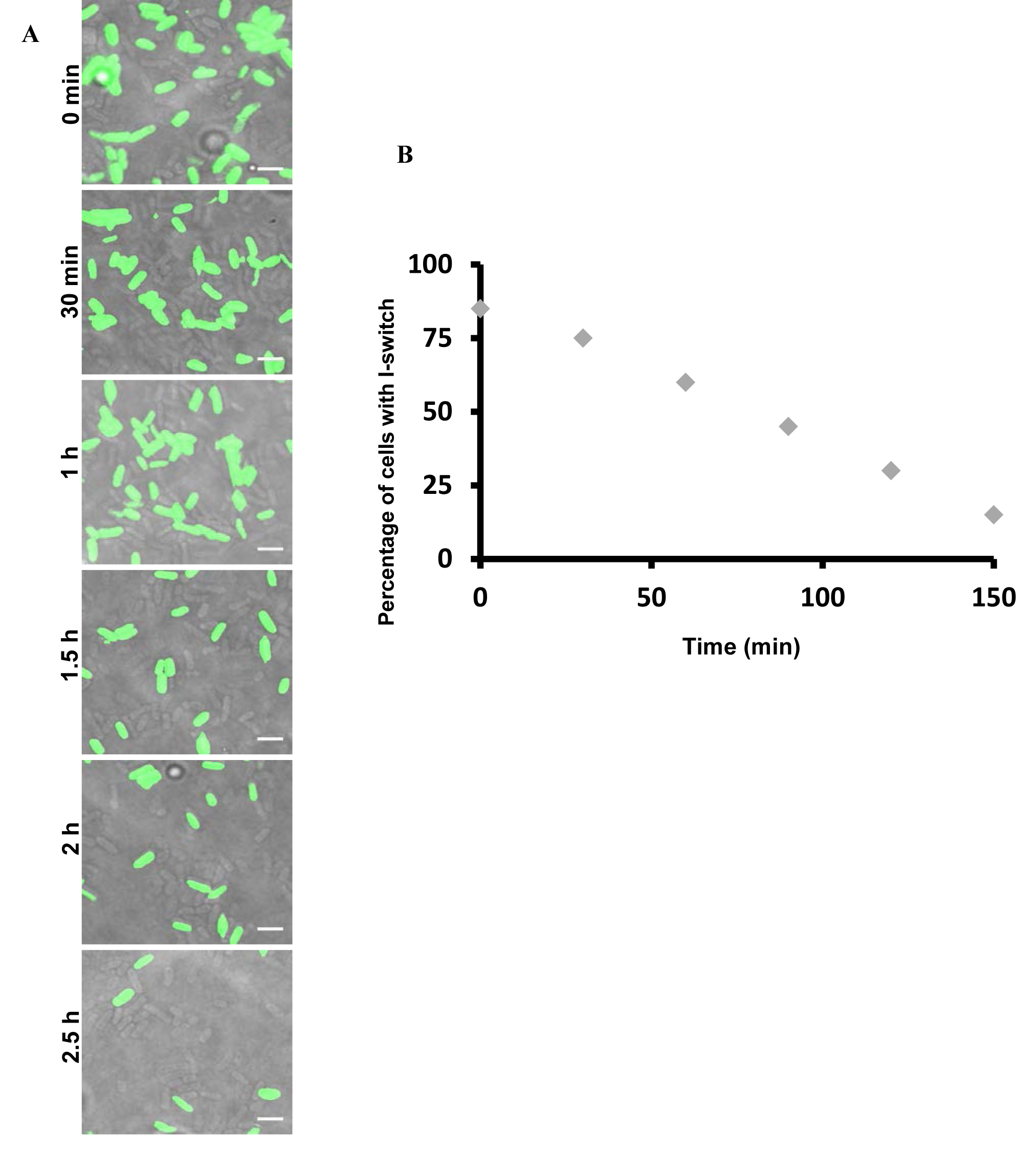

Supplement: S12 Fig — (A) Single labeled I-switch (IA647) incorporated WT Salmonella was incubated in MgM pH 5.6 for 2.5 h. Representative merged images of phase contrast and epifluorescence of the acceptor channel (green) were obtained on an Applied Delta Vision wide field fluorescence microscope every 30 min. Scale bar, 3μm. (B) The percentage of I-switch positive cells is plotted over time. After 2 h of incubation, approximately 20% cells contained visible I-switch. (TIF) [file pbio.1002116.s013.tif]

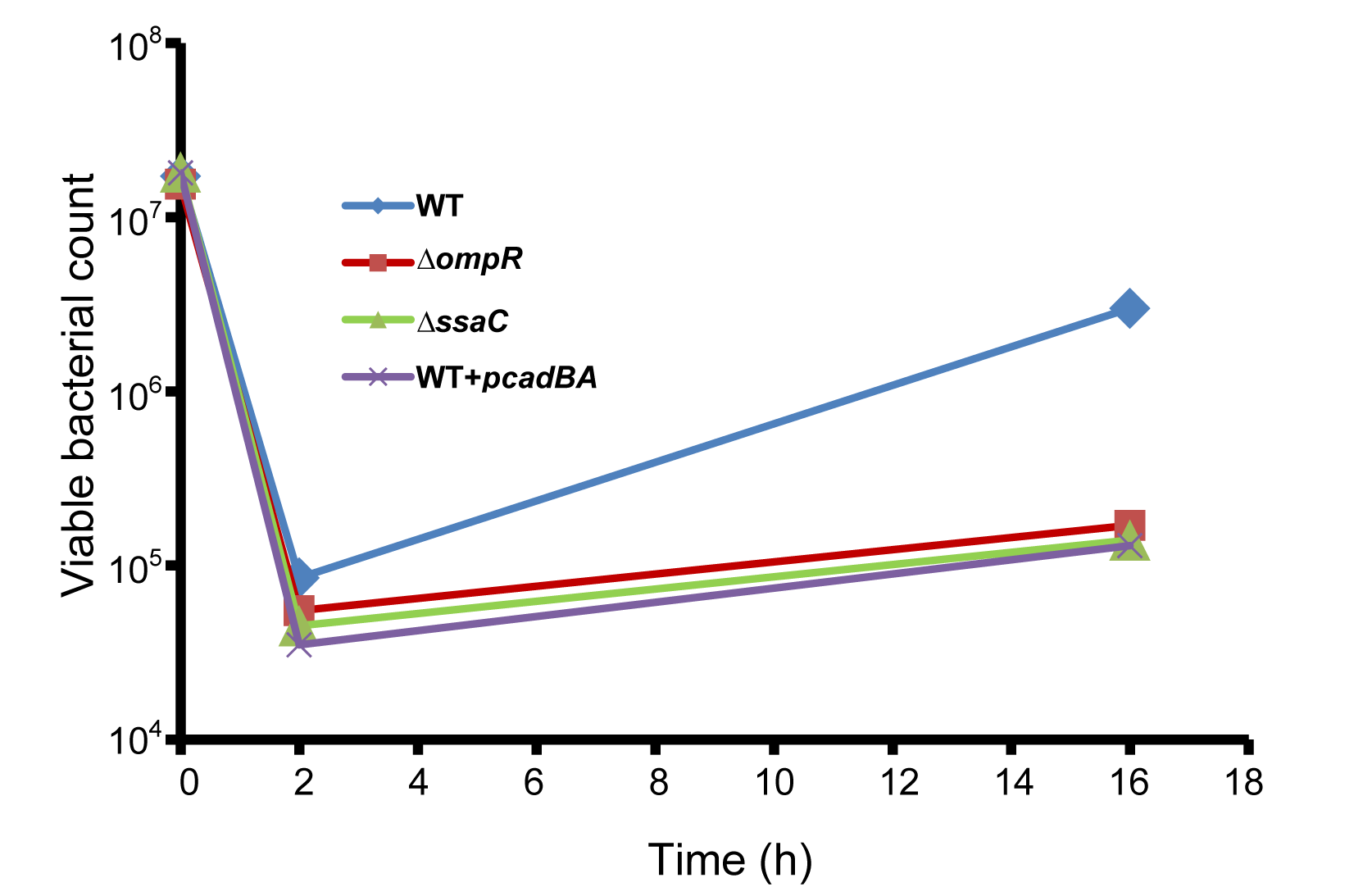

Supplement: S13 Fig — Macrophages were seeded at a density of 105 cells per well in 24-well tissue culture plates, 24 h before use. WT, ompR null, ssaC null and cadBA over-expressed strains of Salmonella were added as described in Materials and Methods. At 2 and 16 h post-infection, host cells were lysed with 0.1% Triton X-100 for 10 min and cultured for enumeration of intracellular bacteria (gentamicin-protected) on to LB agar. All infections were performed in triplicate. The results are represented as the mean ± SEM. (TIF) [file pbio.1002116.s014.tif]

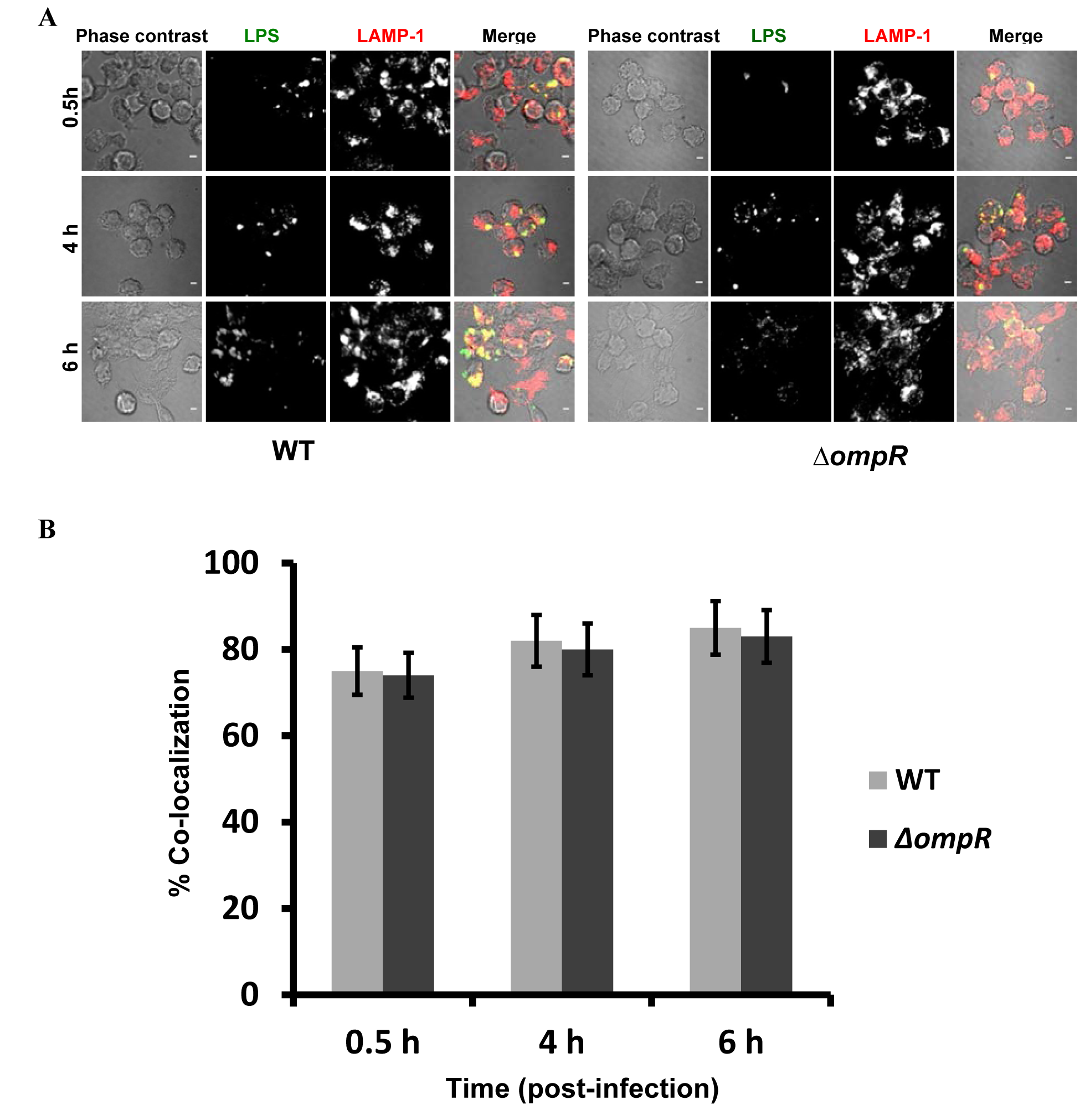

Supplement: S14 Fig — (A) LAMP-1 localizes to the SCV. To determine that Salmonella containing the I-switch were localized to the SCV in macrophages, immuno-labeling was performed with unlabeled I-switch electroporated Salmonella stained with LPS (green) and LAMP-1 (red). The merged image indicates that Salmonella containing the I-switch were localized primarily in the SCVs. Images were obtained using the Nikon A1R confocal microscope. Localization resulted in yellow staining of Salmonella cells as analyzed by Image J software. Scale bar = 3 μm. (B) Results of three independent experiments in which SCVs stained with LAMP-1 were scored for WT Salmonella and an ompC mutant at 0.5, 4, and 6 h post-infection. Co-localization analysis was performed using ImageJ software. The values represent the mean ± SEM. (TIF) [file pbio.1002116.s015.tif]
