# Supplementary material for: A FRET-Based DNA Biosensor Tracks OmpR-Dependent Acidification of Salmonella during Macrophage Infection
Source: PLoS Biol. 2015 Apr 14;13(4):e1002116. doi: 10.1371/journal.pbio.1002116 (PMC4397060; doi:10.1371/journal.pbio.1002116)
Supplement: S1 Table — (DOCX) [file pbio.1002116.s016.docx]

| **Name**  **S1 Table. Sequences of Primers used in this study** | **Sequences (5' to 3')** |
| --- | --- |
| O1 | 5^/^-CCCCAACCCCAATACATTTTACGCCTGGTGCC-3^/^ |
| O2 | 5^/^-CCGACCGCAGGATCCTATAAAACCCCAACCCC-3^/^ |
| O3 | 5^/^-TTATAGGATCCTGCGGTCGG**A**GGCACCAGGCGTAAAATGTA-3^/^ |
| O-488 | 5^/^- Alexa-488-CCCCAACCCCAATACATTTTACGCCTGGTGCC-3^/^ |
| O-647 | 5^/^-CCGACCGCAGGATCCTATAAAACCCCAACCCC-Alexa-647-3^/^ |
| XhoI-P*sseJ* #F | 5^/^-CCGCTCGAGCACTCCCCACGCTATTATGA-3^/^ |
| *sseJ*-HA-BamHI #R | 5^/^-CGGGATCCTTAAGCGTAATCTGGAACATCGTATGGGTATTCAG  TGGAATAATGATGAGC-3^/^ |
| KpnI-P*ompR* #F | 5^/^-GGGGTACCCGGGGTATAACGTGATCGTC-3^/^ |
| *envZ-*HindIII #R | 5^/^-CCCAAGCTTTTATGCCTCTTTTGTCGTCC-3^/^ |
| *sseB*-*tetRA*_in #1F | 5^/-^GGAGATACCGTCAGGAAAAACAAAAAGGTAAAGCATAATGTT  AAGACCCACTTTCACATT-3^/^ |
| *sseB*-*tetRA*_in #1R | 5^/-^GCATCGTGTCATGTGCCTGTTGTAGGGTCGGGTCTTTTTTCTAA  GCACTTGTCTCCTG-3^/^ |
| *cadBA*::*Km* #1F | 5^/-^TAAGCCCGGTTCTTAAAAATACAGCTCAGGAGAAATGAA  CGTGTAGGCTGGAGCTGCTTC-3^/^ |
| *cadBA*::*Km* #1R | 5^/-^CGTGAAAAAAGGGAAGTGGCAAGCCACTTCCCTTTGGTACCTG  TCAAACATGAGAATTAA-3^/^ |
| *cadC*::*Km* #1F | 5^/-^TATTATTAACGTTTGACTTAGCTCGTTAGGGCATCTTTTTGTG  TAGGCTGGAGCTGCTTC-3^/^ |
| *cadC*::*Km* #1R | 5^/-^ATATCTTTTCGCTTTTATTTGTCATGGTTCAGCGTTAAATCTG  TCAAACATGAGAATTAA-3^/^ |
| *cadC* # F | 5^/^-Bio/GCGAAGGGCTGATGGATTTA-3^/^ |
| *cadC* # R | 5^/^-Bio/AAAGATGCCCTAACGAGCTAAG-3^/^ |
| *cadB* # F | 5^/^-Bio/AAATTTAACGCTGAACCATGA-3^/^ |
| *cadB* # R | 5^/^-Bio/GTTCATTTCTCCTGAGCTGTATT-3^/^ |
| *cadA* # F | 5^/^-Bio/CGGTAAGCCTGATTTGTTCTGT-3^/^ |
| *cadA* # R | 5^/^-Bio/CGCCCATGTGGTTCATGATA-3^/^ |
| *envZc* # F | 5^/-^ CGGAATTCCGTATACAGAATCGACCGTT-3^/^ |
| *envZc* # R | 5^/-^  CCCAAGCTTTGCCTCTTTTGTCGTCCCC-3^/^ |
| *ompF:*:*tetRA* #1F | 5^/^-GGCAGGTGTCATATAAAAAAACCAATGAGGGTAATAAATATTAAGACCCACTTTCACATT-3^/^ |
| *ompF*::*tetRA* #1R | 5^/^-CGAAGTCCTGTTTTTGAGGCATAAAACAAAGGGGTCTGCTGACTAAGCACTTGTCTCCTG-3^/^ |
| *ompC*::*Cm* #1F | 5^/^-CATAAAAAAGCAATAAAGGCATATAACAGAGGGTTAATAACCCAGTCTTTCGACTGAGCCT-3^/^ |
| *ompC*::*Cm* #1R | 5^/^-GAAAAAAGGGCCCGCAGGCCCTTTAGCAACATCTTTTGCTGAGAGGCACCAATAACTGCCTT-3^/^ |
| NcoI-*cadBA* #1F | 5^/^-CATGCCATGGGAAGTTCTGTCAAAAAGATCGG-3^/^ |
| *cadBA*-XbaI #1R | 5^/^-GCTCTAGATTATTTCGTATTTTCTTTCAGCAC-3^/^ |
| *mgtC*::*tetRA* #1F | 5^/^-CGTGTGCTAAATATAGCACGTACTTATTCTTCCAGAAAAATTAAGACCCACTTTCACATT-3^/^ |
| *mgtC*::*tetRA* #1R | 5^/^- CTTATACGCCTGGCGTAATGTTGCAATTGAATAAAAAACTACTAAGCACTTGTCTCCTG-3^/^ |
| *mgtC*::*tetRA*_out #1F | 5^/^-CGTGTGCTAAATATAGCACGTACTTATTCTTCCAGAAAAATAGTTTTTTATTCAATTGCAAC-3^/^ |
| *mgtC*::*tetRA*_out #1R | 5^/^-TGAACCCATTGATCAGCAGG-3^/^ |
| *atpB*::*tetRA* #1F | 5^/^-TGGCGCCGGCTGTAATTAACAACAAAGGGTAAAAGGCATCTTAAGACCCACTTTCACATT-3^/^ |
| *atpB*::*tetRA* #1R | 5^/^-CTCCAGTTTGTTTCAGTTAAAACGTAGTAGTGTTGGTAAACTAAGCACTTGTCTCCTG-3^/^ |
| XhoI-P*spiC* #F | 5^/^-CCGCTCGAGAGCGTATTCTTGAGATTGAGCAA-3^/^ |
| *ssaC*-EcoRI #R | 5^/^-CGGAATTCACCTCCGGCACCGCCTGCGCCACCATGAGATATGCCATTAT-3^/^ |
| EcoRI-*gfp* #F | 5^/^-CGGAATTCAGTAAAGGAGAAGAACTTTTC-3^/^ |
| *gfp*-XbaI #R | 5^/^-GCTCTAGATTATTTGTATAGTTCATCCA-3^/^ |
| XhoI-P*sseA* #F | 5^/^- CCCTCGAGAGAAGAGAACAACGGCAAG-3^/^ |
| *sseA*-HindIII #R | 5^/^- CCCAAGCTTCATTCCCCTCCATATACACG-3^/^ |
| HindIII-*ssaJ* #1F | 5^/^- CCCAAGCTTATGAAGGTTCATCGTATAGT-3^/^ |
| mCherry-XbaI #1R | 5^/^- GCTCTAGATTACTTGTACAGCTCGTCCA-3^/^ |
