# Supplementary material for: A FRET-Based DNA Biosensor Tracks OmpR-Dependent Acidification of Salmonella during Macrophage Infection
Source: PLoS Biol. 2015 Apr 14;13(4):e1002116. doi: 10.1371/journal.pbio.1002116 (PMC4397060; doi:10.1371/journal.pbio.1002116)
Supplement: S2 Table — (DOCX) [file pbio.1002116.s017.docx]

| **Strain/plasmid** | **Description** | **Reference/Source** |
| --- | --- | --- |
| **Bacteria** | | |
| 14028s | *Salmonella enterica* serovar Typhimurium |  |
| 14028s ∆*sseB* | 14028s *sseB*::*tetRA* | This study |
| 14028s ∆*ompR* | 14028s *ompR::*Km | [[1](#_ENREF_1)] |
| 14028s ∆*cadA* | 14028s *cadA*:: Km | This study |
| 14028s ∆*cadB* | 14028s *cadB*:: Km | This study |
| 14028s ∆*cadC* | 14028s *cadC*:: Km | This study |
| 14028s ∆*envZ* | 14028s *envZ*:: Cm | Don Walthers |
| 14028s ∆*ssaC* | 14028s *ssaC*::*tetRA* | This study |
| 14028s ∆*ompC* | 14028s *ompC*::Cm | This study |
| 14028s ∆*ompC∆ompF* | 14028s *ompC*::Cm; *ompF::tetRA* | This study |
| 14028s ∆*mgtC* | 14028s *mgtC*::*tetRA* | This study |
| 14028s ∆*atpB* | 14028s *atpB*::*tetRA* | This study |
| **Plasmids** | | |
| pKD46 | λ-Red recombinase expression plasmid, Amp^R^ | [[2](#_ENREF_2)] |
| pWSK29 | Low copy cloning vector, Amp^R^ | [[3](#_ENREF_3)] |
| pMPM-A5Ω | Cloning vector containing paraBAD promoter | [[4](#_ENREF_4)] |
| pBR322 | Cloning vector containing *amp^R^* and *tet^R^* genes | [[5](#_ENREF_5)] |
| pFVP25.1 | GFP mut3 fusion protein | [[6](#_ENREF_6)] |

**S2 Table. Strains and plasmid vectors used in this study**

**References**

1. Feng X, Walthers D, Oropeza R, Kenney LJ (2004) The response regulator SsrB activates transcription and binds to a region overlapping OmpR binding sites at Salmonella pathogenicity island 2. Mol Microbiol 54: 823-835.

2. Datsenko KA, Wanner BL (2000) One-step inactivation of chromosomal genes in Escherichia coli K-12 using PCR products. Proc Natl Acad Sci U S A 97: 6640-6645.

3. Wang RF, Kushner SR (1991) Construction of versatile low-copy-number vectors for cloning, sequencing and gene expression in Escherichia coli. Gene 100: 195-199.

4. Mayer MP (1995) A new set of useful cloning and expression vectors derived from pBlueScript. Gene 163: 41-46.

5. Watson N (1988) A new revision of the sequence of plasmid pBR322. Gene 70: 399-403.

6. Valdivia RH, Falkow S (1996) Bacterial genetics by flow cytometry: rapid isolation of Salmonella typhimurium acid-inducible promoters by differential fluorescence induction. Mol Microbiol 22: 367-378.
